# Evolution of Assortative Mating in a Population Expressing Dominance

**DOI:** 10.1371/journal.pone.0016821

**Published:** 2011-04-01

**Authors:** Kristan A. Schneider, Stephan Peischl

**Affiliations:** Department of Mathematics, University of Vienna, Vienna, Austria; University of Sheffield, United Kingdom

## Abstract

In this article, we study the influence of dominance on the evolution of assortative mating. We perform a population-genetic analysis of a two-locus two-allele model. We consider a quantitative trait that is under a mixture of frequency-independent stabilizing selection and density- and frequency-dependent selection caused by intraspecific competition for a continuum of resources. The trait is determined by a single (ecological) locus and expresses intermediate dominance. The second (modifier) locus determines the degree of assortative mating, which is expressed in females only. Assortative mating is based on similarities in the quantitative trait (‘magic trait’ model). Analytical conditions for the invasion of assortment modifiers are derived in the limit of weak selection and weak assortment. For the full model, extensive numerical iterations are performed to study the global dynamics. This allows us to gain a better understanding of the interaction of the different selective forces. Remarkably, depending on the size of modifier effects, dominance can have different effects on the evolution of assortment. We show that dominance hinders the evolution of assortment if modifier effects are small, but promotes it if modifier effects are large. These findings differ from those in previous work based on adaptive dynamics.

## Introduction

In sexually reproducing populations, mating occurs generally not at random but shows positive or negative correlations with respect to certain characteristics. If pairing of similar males and females is more or less likely than expected by chance, positive or negative assortative mating occurs, respectively. For instance, in humans positive assortative mating has been reported for characteristics such as age, IQ, height, weight, educational and occupational level, and physical and personality characters [1]–[4].

Although assortative mating was studied over the last forty years in the theoretical literature, it received the attention of a much broader audience during the last fifteen years as a possible mechanism leading to sympatric speciation, i.e., speciation without geographical isolation. Classical work focusing on assortative mating studied the mating mechanism itself and kept the strength of assortative mating constant, e.g., [5]–[8]. In the last fifteen years, the evolution of the mating mechanism under a given ecological scenario has been an important topic of research (e.g., [9]–[13]).

Recent studies involving assortative mating were strongly connected to divergence of a quantitative trait within a population or even to sympatric speciation (e.g., [9]–[14]). In these models, a quantitative character is maintained polymorphic by frequency-dependent disruptive selection. Disruptive selection is caused by negative frequency-dependent selection, which was motivated by intraspecific competition for common resources. Assortative mating occurs either with respect to similarities in this ‘ecological’ character (magic-trait model, cf. [15]), or with respect to an additional mating character. The above-mentioned studies used the classical models of resource utilization by Roughgarden [16], Bulmer [17], [18], Slatkin [19], or Christiansen and Loeschcke [20], which all behave similar as long as selection is weak (cf. [21], [22]).

The African finch *Pyrenestes Ostrinus* was often cited to justify the above-described ecological setup (e.g., [23]–[28]). However, assortative mating did not evolve in the African finch. Instead, the finches express dominance (in the ecological character), a mechanism that has been neglected in the above-mentioned studies. (Note that models similar to that in [29] would be more adequate than the above-described approaches, because seeds for which the finches compete are bimodally distributed.)

Recently, some studies focused on finding general conditions for the evolution of assortment [30]–[33]. However, only two attempts were made that explicitly study dominance and assortative mating. The first, by Durinx and van Dooren [30], studied the evolution of assortative mating vs. the evolution of dominance using an adaptive-dynamics approach. The second, by Peischl and Schneider [34], studied the evolution of dominance in an assortatively mating population using a comprehensive numerical approach based on the exact dynamics. Durinx and van Dooren [30] showed that, in the limit of infinitesimally small modifier effects, selection for assortment modifiers is initially stronger than selection for dominance modifiers. Furthermore, they concluded that assortative mating and dominance are alternative and mutually exclusive responses to disruptive selection. In contrast, Peischl and Schneider [34] suggest that the evolution of dominance can be promoted by moderately strong assortative mating. Moreover, they emphasize the importance of the interplay between these evolutionary mechanisms. A necessary step towards understanding the interplay between dominance and assortment is to clarify the influence of dominance on the evolution of assortative mating.

In this article, we study the evolution of assortative mating with respect to an ecological character that expresses dominance. We pursue a population-genetic approach that complements and extends the results of Durinx and van Dooren [30]. We assume an explicit ecological model of frequency-dependent intraspecific competition and assortative mating. Frequency-dependent competition induces indirect selection on a modifier that determines the strength of assortative mating. Dominance relations and the degree of assortative mating control the translation of direct selection at the ecological locus to indirect selection at the modifier locus. In the limit of weak selection, we are able to derive simple invasion conditions for assortment modifiers in a number of interesting scenarios. However, for a fixed combination of parameters, the strength and direction of these effects depend on the genetic distribution of the population and thus vary over time. Hence, for our purpose an invasion analysis is insufficient. Of course, a complete (nonlinear) analysis would be highly desirable, but the complexity of the model prohibits such an analysis. Thus, we pursue a structured and detailed numerical study examining a large part of the parameter space.

We perform a numerical analysis of a two-locus two-allele model, in which the primary (ecological) locus has a major effect on a quantitative trait that is under a mixture of stabilizing selection and frequency-dependent selection caused by intraspecific competition for a continuum of resources. The ecological model follows the one formulated by Bulmer [17], [18]. Moreover, we assume assortative mating. More precisely, females choose mating partners based on similarities in the ecological character. The model of assortative mating used here follows that of Matessi et al. [12], which was originally formulated by Gavrilets and Boake [35]. The secondary locus determines the degree of assortment. In contrast to previous studies of the evolution of assortative mating, we assume that the ecological locus expresses dominance. Our approach is related, but complementary, to that in [34].

Our results show that dominance does not counteract an initial increase of assortative mating. However, the level of assortment that can evolve in small steps is strongly reduced if there is some degree of dominance. By contrast, if modifiers have large effect, dominance can act as a catalyst for the evolution of assortative mating. The parameter region in which strong assortment can evolve is maximized for a certain degree of dominance. Furthermore, this ‘optimal’ degree of dominance increases with increasing modifier effect. We will also discuss the implications of the evolution of assortative mating. If assortative mating is sufficiently strong, divergence within the population occurs. This will eventually lead to sympatric speciation. Dominance can be a mechanism that enforces divergence. Together with the results of a preceding study [34], our results enable us to draw conclusions about the levels of assortment and dominance that are likely to evolve.

### 1.1 The model

We consider a model that is closely related to that in [34]. It assumes a sexually reproducing, diploid, density-regulated population with discrete generations in which both sexes have the same genotype distribution among zygotes. Random genetic drift is neglected by assuming that the population size, 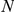, is sufficiently large. Selection acts through differential viabilities on a quantitative character. Because selection is assumed to act on this character, we refer to it as the ‘ecological character’. The viability of an individual is determined by frequency-independent stabilizing selection and by frequency- and density-dependent competition. The trait value of an individual expresses an intermediate degree of dominance. We refer to this trait as the ecological trait. Furthermore, the population mates assortatively with respect to the ecological trait (‘magic trait’). This induces sexual selection. The degree to which an individual mates assortatively depends on its expression at an additional locus that modifies the degree of assortment.

#### 1.1.1 Ecological assumptions

These assumptions follow closely those in [26], [27], [36], where they are motivated. As in most previous studies, we ignore environmental variation and deal directly with the fitnesses of genotypic values. Therefore, we use the terms genotypic value and phenotype synonymously. We denote the ecological trait value of an individual having genotype 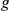 by 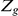.

The frequency-independent fitness component reflects stabilizing selection on the ecological trait, for instance, by differential supply of a resource whose utilization efficiency is phenotype dependent. The stabilizing component acting on genotype 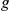 is denoted by 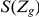. Here, 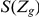 is modeled by the Gaussian function with optimum zero
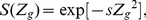
(1)where 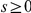 measures the strength of stabilizing selection. We refer to the trait value zero as the position of the optimum or just as the ‘optimum’.

The amount of competition of genotype 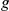 with genotype 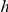 is denoted by 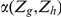. We model it by the Gaussian function
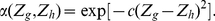
(2)The parameter 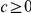 determining the curvature of 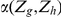 implies that competition between individuals of similar trait value is stronger than between individuals of very different trait value, as it will be the case if different phenotypes preferentially utilize different food resources. Let 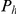 denote the relative frequency of individuals with genotype 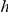. Then the intraspecific competition function 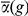, which measures the strength of competition experienced by genotype 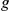 in a population with distribution 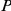, is given by
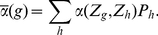
(3)


We include density-dependent population growth, which, in the absence of genetic variation, follows the logistic equation
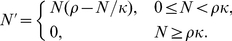
(4)Here, 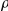 and 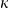 are positive constants, where 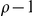 is the intrinsic growth rate and 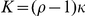, the carrying capacity. Monotone convergence to 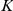 occurs for all 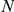 with 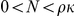 if 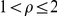, and oscillatory convergence (at a geometric rate) if 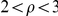. Other forms of population regulation may be used as well (cf. Appendix B in [21]). Following [17], [18], we assume that the absolute fitness of an individual with genotype 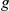 is
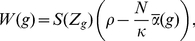
(5)where the dependence of 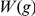 on 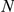 and 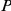 is omitted. Although 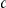 is a direct measure for the strength of the frequency-dependent effect of competition, rather than of competition itself, for convenience, we shall refer to 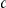 as the strength of competition.

In part of this work we will replace (1) and (2) by the corresponding quadratic approximations, i.e., by
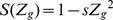
(6)and
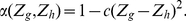
(7)In addition, we will assume a constant population size close to the demographic equilibrium. Then fitness of an individual with genotype 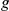 is given by
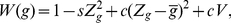
(8)where 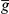 is the mean and 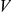 the variance of the phenotype distribution (cf. [21]). As long as the mean genotypic value is sufficiently close to zero, 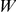 is 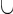-shaped if and only if 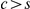 and 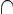-shaped if and only if 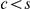. We will refer to (5) with 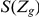 and 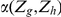 given by (1) and (2), or by (6) and (7) as the *Gaussian model* or the *quadratic model*, respectively. Note that the quadratic model can be regarded as the weak-selection approximation of the Gaussian model, i.e., as an approximation for small 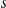 and 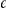.

In [37], the quadratic model was used to study the evolution of dominance in a randomly mating population. This weak-selection approximation is also used in [26], [27] to study closely related ecological models under different assumptions and with another focus. The Gaussian choice has the advantage that weak and strong selection can be modeled, but it is prohibitive to a general mathematical analysis. For our numerical results investigations we will always assume the Gaussian model, whereas, unless otherwise specified, we will use the quadratic model for our analytical results.

#### 1.1.2 Assortative mating

We assume that mating is assortative according to the model of Matessi et al. [12], which is a particular case of the model introduced by Gavrilets and Boake [35]. The probability that a random encounter between a female and a male results in mating depends on similarities in the ecological character (‘magic trait’). More precisely, the probability that at a given encounter, a 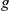-female mates an 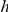-male is given by 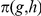, and modeled by
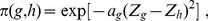
(9)where 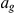 is the strength of assortment expressed by a female with genotype 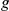. In fact, 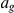 depends only on the modifier locus and is a direct measure for the strength of assortative mating. Note that 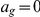 means that a female mates randomly, whereas 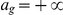 means that she mates only males that show an identical value of the ecological trait. In this article, we always assume 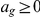, i.e., we consider only positive assortative mating.

Females are assumed to mate only once, whereas males may participate in multiple matings. If an encounter was not successful, in which case she remains unmated, she may try again unless she successfully mated. Hence, the probability that a female mates successfully equals one, implying no costs for choosiness. The probability that an encounter of a 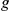-female with a random male results in mating is
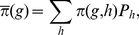
(10)and the probability that she eventually mates with an 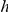-male is calculated to be 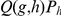, where
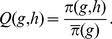
(11)Here, the first argument refers to the female. Note that in general 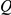 is not symmetric in 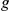 and 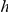.

Limitations on the total number of unsuccessful mating attempts of females would imply costs for choosiness. See [27] for more discussion. They also derive the above equations as the limiting case in which the number of possible mating attempts reach infinity (cf. also [34]).

Although, there are no costs for female choosiness, there are costs for rare males, because they are less likely to mate successfully. In other words, common males mate more often than rare males.

#### 1.1.3 Genetic assumptions

Regarding the underlying genetics, we assume that the ecological trait is determined by a single diallelic locus. We denote the alleles segregating at this locus by 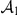 and 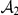, and their effects by 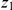 and 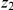, respectively, which we assume to be symmetric, i.e., 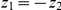. We make this assumption, on the one hand, to minimize complexity of the model, and on the other hand, to keep computational time for our numerical investigations manageable (see Discussion for possible implications if this assumption is waived). By rescaling the parameters 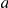, 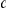, and 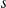 we can assume without loss of generality that
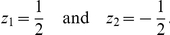
Moreover, 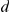 is the degree of dominance. Hence, individuals with the allele configurations 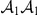, 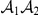, and 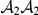 at the ecological locus, have trait values 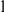, 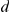, and 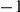, respectively. Here, we consider only intermediate dominance, i.e., 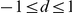. Clearly, 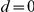, 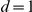, or 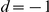 means no dominance, complete dominance of 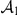, or complete dominance of 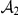, respectively. The symmetry assumption implies that we can assume 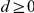 without loss of generality.

The strength of assortment expressed by females is determined by a separate diallelic, autosomal locus (“modifier locus”). The two alleles at this locus are denoted by 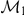 and 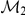. The alleles have effects 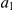 and 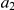, respectively, which additively determine the strength of assortment expressed by females. Hence, a female carrying the allele combination 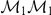, 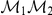, or 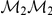 expresses assortment at strength
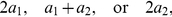
(12)respectively.

Whenever we refer to modifiers increasing assortment, we call the allele at the modifier locus that codes for a higher level of assortment the mutant or the modifier allele, and the allele coding for a lower level of assortment the wild-type allele. In the case of modifiers decreasing assortment, it is the other way round. The initial strength of assortment, a, refers to the degree of assortment expressed by individuals that carry the wild-type allele homozygous at the modifier locus. The difference between the initial degree of assortment and the degree of assortment expressed by individuals that are heterozygous at the modifier locus is the effect of the modifier allele, 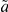. In other words, if 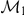 is the wild-type allele, then
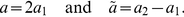
(13)


#### 1.1.4 Dynamics

The two-locus dynamics has to be described in terms of diploid genotype frequencies since zygotes (offspring) are generally not in Hardy-Weinberg proportions because of assortative mating. Genotypes are unordered. Let 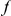 represent an offspring's genotype and 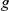, 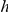 parental genotypes. The frequencies of genotype 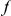 (among zygotes) in consecutive generations are denoted by 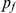 and 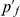. The frequency of 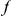 after (natural) selection is 
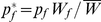
, where 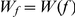 and 
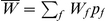
 is the mean viability. After viability selection, mating and recombination occur. Let 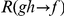 designate the probability that parents with genotypes 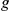 and 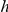 produce a zygote with genotype 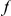. 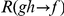 is determined by the pattern of recombination between the two loci. The recombination rate between the two loci is denoted by 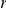.

The genetic dynamics is given by a system of 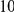 recursion equations that can be written as
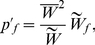
(14)where
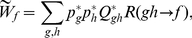
(15)

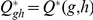
 (the asterisk indicates that 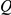 is calculated from the genotypic frequencies after selection) and 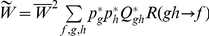. The demographic dynamics follows the standard recursion
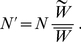
(16)Thus, for a genetically monomorphic population that matches the optimum, population growth follows (4). The complete evolutionary dynamics is given by the coupled system (14) and (16). We set 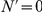 (population extinction) if 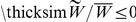, and 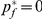 if 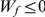.

In the quadratic model, population size is assumed constant and the dynamics is given by (14).

### 1.2 Components of selection

Before we start describing our methods and results, we discuss the different selection pressures and their effect on selection at the modifier locus.

Modifier alleles affect the strength of assortative mating but not the phenotypic value of an individual that carries the modifier. In addition, we assume that modifiers do not have a direct fitness effect. This means that direct selection on the ecological locus is translated to indirect selection at the modifier locus. An increase in the strength of assortment leads to a decrease in the frequency of heterozygotes at the ecological locus. Therefore, higher levels of assortment are favored if heterozygotes are, on average, less fit than homozygotes [12], [13], [30], [31]. We call the net effect of selection disruptive if heterozygotes (at the ecological locus) are less fit than homozygotes, and stabilizing if heterozygotes are fitter than homozygotes. The strength of selection at the modifier locus depends on the frequency of heterozygotes at the ecological locus. Selection is transmitted more efficiently if the frequency of heterozygotes is high. If the frequency of heterozygotes goes to zero, selection at the modifier locus vanishes.

Selection acts directly at the ecological locus via four components. The first component of selection in our model is frequency-independent stabilizing selection. We assume symmetric allelic effects with respect to the optimum of stabilizing selection (for a discussion of this assumption see [34]). Thus, phenotypes close to the middle of the phenotypic range are favored by stabilizing selection. This leads to heterozygote advantage and selection against modifiers that increase assortment. Since we assume symmetric allelic effects, heterozygote advantage is strongest in the absence of dominance. In the numerical part of this work, we only consider stabilizing selection that is weak compared to negative frequency-dependent selection (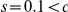).

The second component is negative frequency-dependent selection induced by intraspecific competition. It favors sufficiently different phenotypes such that competition between individuals is minimized. We interpret these phenotypes as being adapted to different ecological niches, where we interpret the location of the maxima of 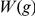 (given either by eq. 5 or eq. 8) as ecological niches. We focus on at most moderately strong competition. Then 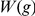 is 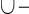
shaped in the absence of dominance and assortative mating, i.e., 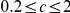. If 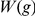 is 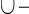
shaped, two ecological niches exist, coinciding with the phenotypic values of the homozygotes, i.e., −1 and +1. In this situation, intraspecific competition favors an increase in genetic variance and therefore higher levels of assortment. However, assortment may change the shape of 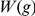. If heterozygotes are rare because of assortative mating, a niche in the middle of the phenotypic range can be established, which can lead to selection for lower levels of assortment. Dominance generally decreases the difference in viability between homozygotes and heterozygotes. This weakens indirect selection at the modifier locus.

The third component, density-dependent selection, acts jointly with intraspecific competition. For a given population distribution, the fitness ratio of advantageous to disadvantageous phenotypes is larger in high-density than in low-density populations.

The forth component is positive frequency-dependent selection induced by assortative mating. Positive frequency-dependence favors common types over rare types. Hence, it counteracts intraspecific competition in this sense. Although we assume no costs of choosiness, the disadvantage of low-frequency males can be interpreted as costs of being rare. Hence, positive frequency-dependence is stabilizing if heterozygotes are common and disruptive if heterozygotes are rare. The difference in the mating success of heterozygotes and homozygotes determines whether higher or lower levels of assortment are favored by positive frequency-dependent selection. Thus, weak initial assortment favors a decrease in the strength of assortment, and strong initial assortment favors an increase in the strength of assortment. However, the strength of assortment also determines the efficiency of indirect selection. If sexual selection is strong because of high levels of assortment, indirect selection at the modifier locus may nevertheless be very weak because of a reduced frequency of heterozygotes at the ecological locus. In addition, dominance decreases the difference in mating success between homozygotes and heterozygotes, and thus the strength of selection at the modifier locus.

## Methods

For a detailed derivation of the analytical results we refer to Appendix S1. A detailed mathematical analysis of our model beyond the analytical results presented in Section 3.2 seems infeasible. Thus, we additionally pursued a comprehensive numerical analysis. Our numerical approach consists of two parts.

In the first part, we numerically calculated the invasion fitness of an initially rare modifier of effect 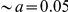 in a population close to equilibrium for several values of 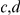, and 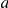 (see Figure 2). (The equilibrium was found by numerically iterating the (14) and (16) from ten different initial frequencies. All trajectories always converged to the same equilibrium.) By invasion fitness we mean the leading eigenvalue of the linearized transition matrix described in Appendix S1. Invasion fitness helps us to identify regions in which higher levels of assortment are favorable if the modifier locus is fixed for the wild-type allele.

Our main focus is the second part, where we obtain a complete picture of the global dynamics by performing numerical iterations of the coupled system (14) and (16). For the iterations, we performed three sets of calculations. In the first set, the assortment modifier was assumed to initially segregate at random frequency in the population. In particular, the genotype frequencies are drawn from a uniform distribution and then normalized. In the second set, we assumed that the assortment modifier is initially rare, i.e., at frequency 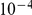. Furthermore, we assumed that initially the genotypes 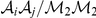 (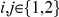) were not present. In the third set, the assortment modifier was assumed to initially segregate at high frequency. We proceeded analogously to the second scenario, but the initial frequency of the modifier allele was 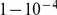. For simplicity, we call the first set of iterations the *standard* scenario, the second situation the *rare-modifier* scenario, and the third situation the *frequent-modifier* scenario.

Throughout our numerical investigations we assumed free recombination, i.e., the recombination rate was 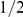, and we always chose the population growth rate to be 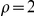. Moreover, because 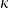 can be considered a scaling factor for the population size 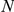, we did not choose it explicitly, and instead regarded 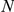 as normalized by the carrying capacity. We assumed that the initial population size matches exactly the carrying capacity, i.e., 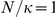.

Our model is fully determined by the parameter vector 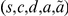. In all scenarios we used 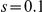. The other parameters were varied as described below. Moreover, we chose various values for 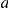 and 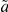 that are listed in the figure captions and in the description of our results. For each combination of the above parameters, we chose ten different initial genotype distributions under all three scenarios, subject to the constraint that the minimum Euclidean distance between any two different distributions is 0.2.

For each initial distribution, we iterated the recursion relations (14) and (16) either until an equilibrium was reached, which was decided to be the case if the Euclidean distance between the vectors of genotype frequencies concatenated with the population size of two consecutive generations was less than 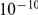, or until 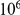 generations were reached. The latter are referred to as *slow runs*. The reason was always slow convergence to equilibrium, not cyclical or chaotic behavior.

## Results

The net impact of the different selection components on the modifier locus depends crucially on the combination of parameters. In general, competition and sexual selection act in opposite directions but it is not straightforward to determine how they interfere in detail. For instance, the net effect of selection can be disruptive although either sexual or natural selection is stabilizing. In addition, dominance can have a strong effect on the strength and direction of selection at the modifier locus.

**Figure 1:**
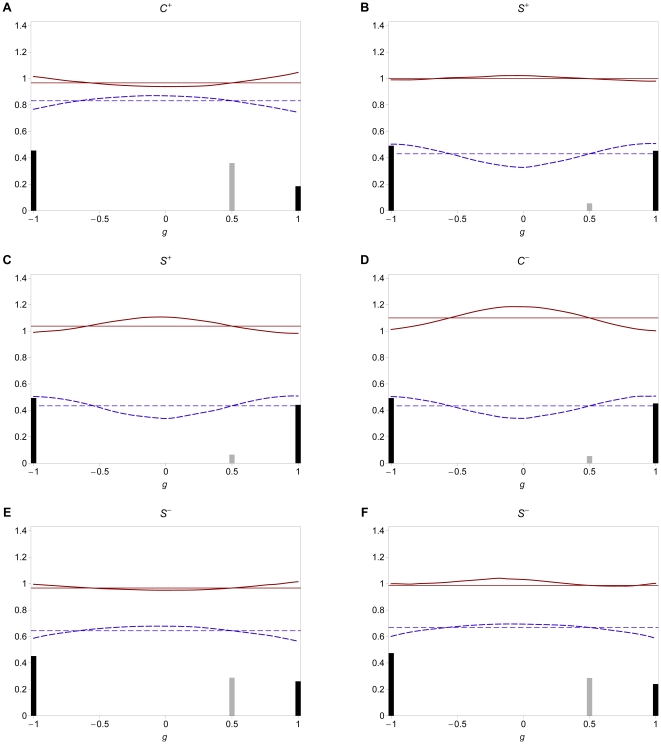
Viability and mating success for different regimes. Viability 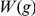 (thick solid line; cf. eq. 5), mating probability 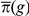 (thick dashed line) and phenotype distributions (black and gray bars) at equilibrium in the different scenarios described in Resluts. Thin straight lines show the viability (solid) and the mating probability (dashed) of heterozygotes. Equilibrium frequencies of homozygotes on the ecological locus are indicated by black bars and frequencies of heterozygotes are indicated by gray bars. Parameter values are (A) 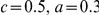, (B) 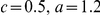, (C) 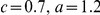, (D) 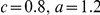, (E) 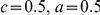, and (F) 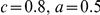. The other parameters are 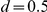, 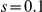, and 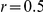 in all figures.

We encountered four dominating selection regimes in our analysis (cf. Figure 1). Clearly, an increase in assortative mating always reduces the number of intermediate phenotypes. Roughly speaking, higher levels of assortative mating evolve only if heterozygotes, i.e., intermediate phenotypes, are deleterious.

Intermediate phenotypes (heterozygotes) are common as long as assortment is weak. If negative frequency-dependent selection (resulting from competition) outweighs positive frequency-dependent selection (resulting from assortative mating), heterozygotes are deleterious due to strong competition and higher levels of assortment can evolve (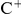 regime, cf. Figure 1A). However, if the reverse is true, intermediate phenotypes are advantageous because they are more likely to participate in successful matings. Hence higher levels of assortment cannot evolve (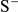 regime, cf. Figure 1E and F).

For stronger assortment, heterozygotes are rare. Intermediate phenotypes might be advantageous due to reduced competition (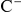 regime, cf. Figure 1D), such that stronger assortment cannot evolve. However, heterozygotes might also be deleterious because they participate less in successful matings due to their reduced frequency (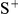 regime, cf. Figure 1B and C).

In our terminology ‘C’ stands for competition, and ‘S’ for sexual selection due to assortative mating. The superscripts ‘
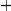
’ and ‘
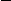
’ indicate selection for higher or lower levels of assortment, respectively. The direction of selection at the modifier locus was determined by numerically calculating the rate of change of modifier alleles. We will describe the selective regimes in more detail when we present our numerical results.

Noteworthy, in the quadratic model, the 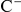 regime is impossible if 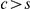. Namely, the frequency of heterozygotes at the ecological locus only changes the intensity of disruptive competition, but not the 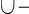
shape of viability. This reflects a very important difference between the quadratic and the Gaussian model.

We first present numerical results on the invasion fitness, followed by analytical results, to acquire a basic understanding of the dynamics. This will help to understand our numerical investigations of the global dynamics.

### 3.1 Invasion fitness

**Figure 2:**
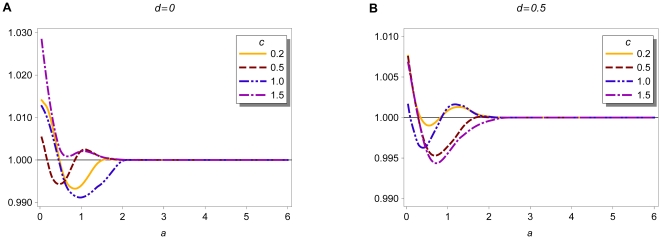
Invasion fitness. Invasion fitness as a function of the initial degree of assortment for various values of 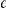 and 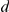. In A, 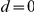 and in B, 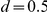. The modifier effect is 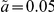 in both figures. Furthermore, we used 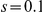 and 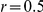. The invasion fitness was calculated at 20 equidistant points in the interval 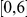.

We numerically calculate the invasion fitness of an initially rare modifier. Figure 2 shows the invasion fitness of a modifier with small effect (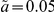) as a function of the initial level of assortment in the absence of dominance (A), and for 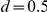 (B). We note that all results are qualitatively robust with respect to the size of the modifier effect.

First, we consider no dominance, i.e., 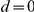 (Figure 2A). For weak assortment (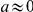), a modifier that increases the degree of assortment can always invade. The reason is that (5) is 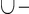
shaped in the considered parameter region and intraspecific competition dominates over assortative mating (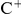). If initial assortment increases, positive frequency-dependence increases and disruptive selection at the ecological locus is weakened. Provided competition is weak (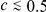) and assortment is weak or moderate (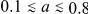), intermediate phenotypes (heterozygotes) participate more often in successful matings. Hence, they are advantageous and stronger assortment cannot evolve (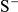). In this region, positive frequency-dependence outweighs negative frequency-dependence and selection is ‘overall’ stabilizing. If both competition and assortment are moderately strong (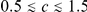, 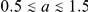), the number of heterozygotes is sufficiently low such that they become advantageous due to reduced competition (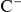), and a modifier that increases assortment cannot invade. Apparently, 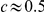 is optimal for the evolution of assortment in small steps. Then, the 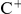 regime applies if 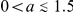. If initial assortment is high (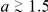), modifiers are almost neutral and the 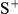 regime applies if 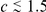. If 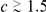, disruptive sexual selection cannot outweigh the viability advantage of heterozygotes at the ecological locus. Thus, the 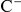 regime applies. In general, selection for modifiers is very weak if 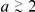. This is because the selective strength at the modifier locus depends on the frequency of heterozygotes at the ecological locus, which is very low for high levels of initial assortment.

Next, we consider intermediate dominance, 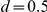 (Figure 2B). Selection at the modifier locus is in general weaker. The reason is that heterozygotes resemble one of the homozygotes more closely. Hence, the fitness difference between heterozygotes and homozygotes is smaller, which leads to weaker selection at the modifier locus. In addition, the narrow region in which assortment can evolve in (‘infinitesimally’) small steps (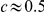) vanishes in the presence of dominance. If 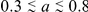, dominance decreases disruptive competition at the ecological locus more strongly than the differences in mating success between homozygotes and heterozygotes. Therefore, the 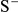 regime applies and assortment cannot further evolve. Dominance has no significant effect on invasion fitness if assortment is sufficiently strong (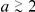). Then, the 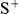 (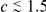), or 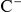 (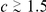) regime applies and selection at the modifier locus is very weak. These findings suggest that dominance hinders the build-up of reproductive isolation in small steps.

Although the concept of invasion fitness is a useful first step in understanding the evolutionary dynamics, to clarify the global dynamics more information is needed. Together with the other parameters, the degree of assortment determines, which of the regimes described above applies. Since assortment evolves in our model, different regimes can apply at different points in time for a fixed set of parameters. Our analytical results on the evolution of assortment show that the build-up of reproductive isolation is most likely if modifier alleles have large effects (see also [12], [13], [30], [31]). However, predictions based on invasion fitness are most accurate for small modifier effects. Thus, it is necessary to consider the global dynamics of the model to gain complete understanding of the effect of dominance on the evolution of assortative mating. However, we shall first present analytical results that will improve our intuitive understanding for the global dynamics.

### 3.2 Analytical results

To derive analytical results we use the quadratic model (8) and assume a population of constant size close to demographic equilibrium. In addition, whenever we speak of weak assortment, we choose the probability that a 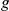-female mates an 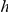-male at a given encounter as
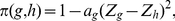
(17)i.e., the first-order Taylor approximation in 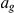 of (9) around 0. This imposes the restriction 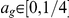. (Note that we also have the restriction 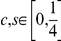 in the quadratic model.)

Throughout this section, we assume that the population is at an equilibrium at which the modifier locus is monomorphic and the ecological locus is polymorphic (cf. [34]). The state of the population is then perturbed by the occurrence of a modifier allele at low frequency. We present invasion criteria for such modifiers in various scenarios. We derive these conditions by calculating (approximations for) the leading eigenvalue of the linearized transition matrix of the gene-frequency vector at equilibrium, i.e., we perform a local stability analysis. Equilibria can be calculated explicitly only if dominance is complete or absent, and if the population mates either randomly or completely assortatively. However, by using standard perturbation techniques, approximations for the equilibria and their eigenvalues can be derived in a number of interesting cases such as weak or strong initial assortment, and weak or strong dominance. The equilibria and the derivations of the following results are given in Appendix S1.

#### 3.2.1 No dominance

The case of no dominance is the simplest and has previously been treated in the literature in a number of similar but different models [12], [13], [30]. In the absence of dominance, we restrict attention to symmetric equilibria, i.e., we assume that both homozygotes at the ecological locus have the same frequency (see Appendix S1 for a justification of this assumption).

1. Modifiers with small effects: By small effect we mean that 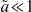, so that we can neglect second and higher order terms in 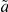. The assumption of no dominance and small modifier effects allows us to use an invasion criterion derived in [12]. Useful application of this criterion requires explicit knowledge of genotype frequencies at equilibrium. Additionally, we also derive approximations for the leading eigenvalues. This gives us an estimate of the strength of selection on a rare modifier allele.

Weak initial assortment: We address three questions. First, when will a modifier inducing a small degree of assortment invade a randomly mating population? Second, when will it go to fixation provided it is sufficiently frequent? Third, when can a modifier invade a population that already expresses a small degree of initial assortative mating?

Let 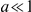 so that we can use (17). We show in Appendix S1 that a modifier increasing assortment invades the population at equilibrium if and only if
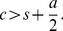
(18)Hence, in a randomly mating population (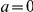) a modifier invades if and only if 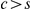. Furthermore, a modifier that decreases assortment can invade if and only if the inequality in (18) is reversed.

The above implies that a sufficiently frequent modifier that increases assortment becomes fixed if and only if
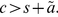
(19)


Strong initial assortment: If the population expresses strong assortment, i.e., if 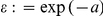 is sufficiently small to neglect terms of order 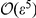 and higher, it is possible to derive conditions for the spread of modifiers slightly increasing the strength of assortment. In contrast to the case of weak initial assortment, modifiers can always invade a strongly assortatively mating population. Furthermore, a modifier with (‘infinitesimally’) small effect 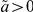 will go to fixation provided it is sufficiently frequent. Hence, modifiers that decrease the strength of assortment cannot invade if a sufficiently high level of assortment is established.

Concluding, a modifier inducing a small degree of assortment invades a randomly mating population if and only if selection is disruptive, i.e., 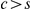 (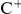 regime). The modifier may however not be able to go to fixation. This is the case if 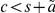 (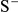 regime). Hence, the individuals in the population will express different degrees of assortment. However, if the modifier goes to fixation, disruptive selection is sufficiently strong and a new modifier that increases assortment can invade. If assortment is sufficiently strong, modifiers increasing assortment will always invade if rare, and go to fixation if sufficiently frequent (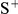 regime).

2. Large modifier effects, initial random mating: As shown in Appendix S1, a modifier that increases assortment can invade a randomly mating population if and only if 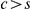, independently of the size of the modifier effect. In fact, the invasion condition does not change even for arbitrary mate-choice functions that induce positive assortment. This includes the case of a modifier that causes individuals that carry at least one copy of the modifier to mate completely assortatively, i.e., if 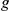, 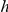 have the form 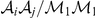, 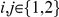, we set 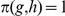 and otherwise
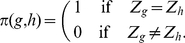
(20)


Furthermore, modifiers with sufficiently large effect always go to fixation if they are sufficiently frequent (regime 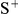).

To summarize, in the absence of dominance, modifiers with small effects can invade a randomly mating population, but may not be able to get fixed. In contrast, modifiers with large effect can invade whenever selection is disruptive, and, in addition, they go to fixation if they are sufficiently frequent. Thus, for an initially randomly mating population, we conclude that complete reproductive isolation is most likely to evolve in large steps if there is no dominance.

#### 3.2.2 Weak or strong dominance, random mating

How does dominance affect the evolution of assortative mating? Analytical results in models with dominance are difficult to obtain and hence rare in the literature. In our model, three cases are analytically tractable to some extent, namely random mating and weak or strong dominance, and complete assortment and arbitrary (intermediate) dominance. The invasion criterion for modifiers of small effect cannot be used in the case of dominance. Instead, we have to calculate approximations for the leading eigenvalues.

Weak dominance: Let dominance be sufficiently weak to neglect terms of order 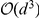 and higher (see Appendix S1). In this case, the leading eigenvalue of the linearized transition matrix is
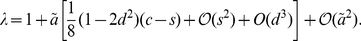
(21)Hence, a modifier can invade if and only if 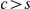. Although the strength of selection for a modifier is a decreasing function in 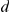, the invasion criterion is not affected by weak dominance.

Strong dominance: Let 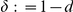 and assume that terms of order 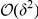 and higher can be neglected (see Appendix S1). The leading eigenvalue is
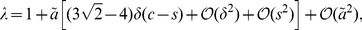
(22)and a modifier increasing assortment can invade if 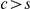. Note, that the invasion fitness is again a decreasing function in 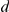. In the case of complete dominance, 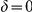, modifiers for assortative mating are selectively neutral and the leading eigenvalues equals 1. This can easily be generalized to modifiers with arbitrary effect.

The above results suggest that dominance decreases the strength of selection for rare assortment modifiers, but has no effect on the condition for invasion (cf. [30]), at least for weak or strong dominance. This is of course only true in the deterministic model. In a stochastic version dominance would also decrease the probability of successful invasion.

Clearly, (21) and (22) imply that the invasion fitness becomes higher as the frequency-dependent effect of competition increases, ie., larger 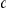. Moreover, for small 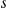 and 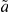 (21) becomes approximately 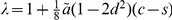 for weak dominance, and (22) becomes approximately 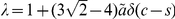 for strong dominance. In particular, as intuitively expected, modifiers become almost selectively neutral for high levels of dominance. Therefore, invasion fitness seems to be a decreasing function of the level of dominance. The decrease in invasion fitness is not linear and (21) even suggest that modifiers might not be able to invade if dominance is intermediate. However, neither (21) nor (22) is a good approximation for intermediate levels of dominance, and conclusions on this case cannot be drawn. Typical, for the quadratic model is the condition 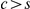. The fitness changes from stabilizing to disruptive as 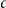 becomes larger than 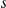 (cf. [21], [22], [36]).

#### 3.2.3 Complete Assortment

Intermediate dominance and complete assortment: Suppose dominance is intermediate, i.e., 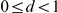 (which includes the case of no dominance) but otherwise arbitrary. Furthermore, assume that the population mates completely assortatively. Then, a unique polymorphic equilibrium exists (see Appendix S1). Consider an initially rare modifier that decreases the strength of assortment by an arbitrary amount. In Appendix S1, we show that such a modifier can never invade, as long as the modifier leads to a positive mating probability between the homozygotes (at the ecological locus). (Note that invasion of such a modifier would imply that complete assortment could not be achieved by small steps.) If the mating probability between homozygotes is zero, a rare modifier decreasing assortment is neutral.

Complete dominance and complete assortment: In Appendix S1, we show that modifiers decreasing assortment by an arbitrary degree are selectively neutral in populations in which dominance and assortment are initially complete. The same holds for modifiers decreasing dominance by an arbitrary degree.

#### 3.2.4 Assortment vs. dominance

Here, we compare the (initial) strength of selection for an increased level of assortment with the selection pressure for an increased level of dominance. The strength of selection for a rare dominance modifier in a randomly mating population for the same ecological model is given in [34]. Hence, we can compare the strength of selection for the different modifiers. If the modifier effects go to zero, the selection coefficients for a dominance modifier and an assortment modifier behave differently (see Appendix S1). The strength of selection for a dominance modifier decreases faster than the strength of selection for an assortment modifier. This is consistent with previous results [30] that showed that in symmetric cases selection for an increased level of assortment is stronger than selection for an increased level of dominance if both modifiers have infinitesimally small effects.

### 3.3 Numerical results on the global dynamics

Here, we consider the complete evolutionary trajectory of the gene-frequency vector and the population size. A newly introduced modifier can either rise to fixation, die out, or can be maintained at intermediate frequency. Furthermore, the existence of multiple stable equilibria is possible. Consequently, the fate of a modifier may depend on its initial frequency.

#### 3.3.1 Invasion, maintenance and fixation of a modifier with small effect

First, we consider modifiers of small effect (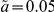) in an initially randomly mating population. The impact of the modifier's effect size is discussed in Section 3.3.2, and that of the initial degree of assortment in Section 3.3.3.

**Figure 3:**
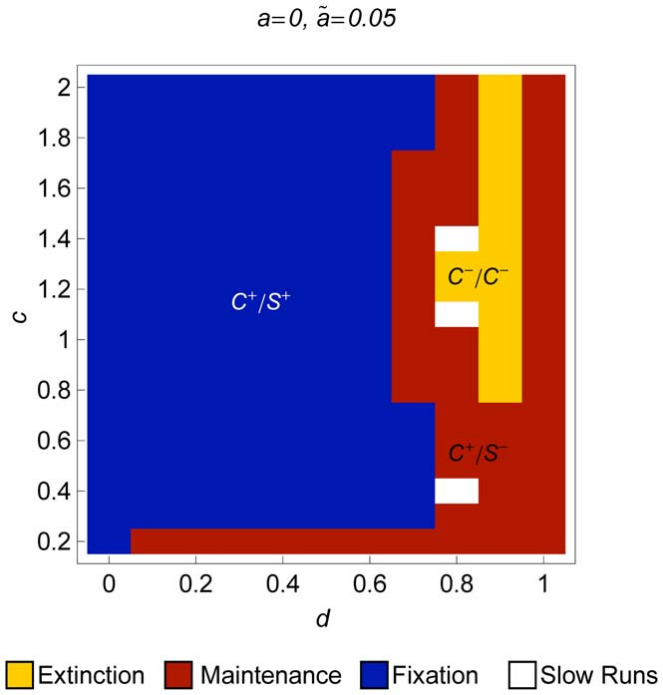
Extinction/maintenance/fixation of small modifiers in an initially randomly mating population. Regions of extinction, maintenance, and fixation of a modifier increasing assortment slightly (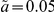) in an initially randomly mating population. We used a grid with stepsize 0.1 for the parameters 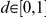 and 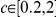. The other parameters are 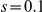 and 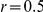. In addition to the color code, different regions are labeled 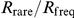, where 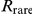 and 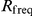 are the selection regimes that apply if the modifier is rare or frequent, respectively. The color code indicates the different evolutionary outcomes. In the extinction regions, the modifier died out in all runs. In the maintenance regions, the modifier coexisted with the wild type in all runs, whereas in the fixation region the modifier was fixed for all runs. Parameter combinations for which none of the runs equilibrated within 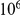 generations are indicated as slow run regions.

Figure 3 illustrates the evolutionary outcome for a modifier with effect 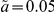. Multiple stable equilibria were not detected, except for complete dominance (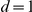). Thus for 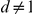, all results apply for the standard, rare-modifier, and frequent-modifier scenario. For 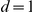 there seems to exist a manifold of equilibria, at which both phenotypes are equally frequent. All trajectories converged to a different equilibrium dependent on the initial conditions. (For an initially randomly or completely assortatively mating population the invasion fitness of modifiers equals one, i.e., they are neutral, see Section 3.2.2. In Figures 3–6, the regions with 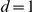 are marked as regions of maintenance. Modifier that are initially at low frequency or high frequency, will neither get lost nor become fixed.)

**Figure 4 pone-0016821-g004:**
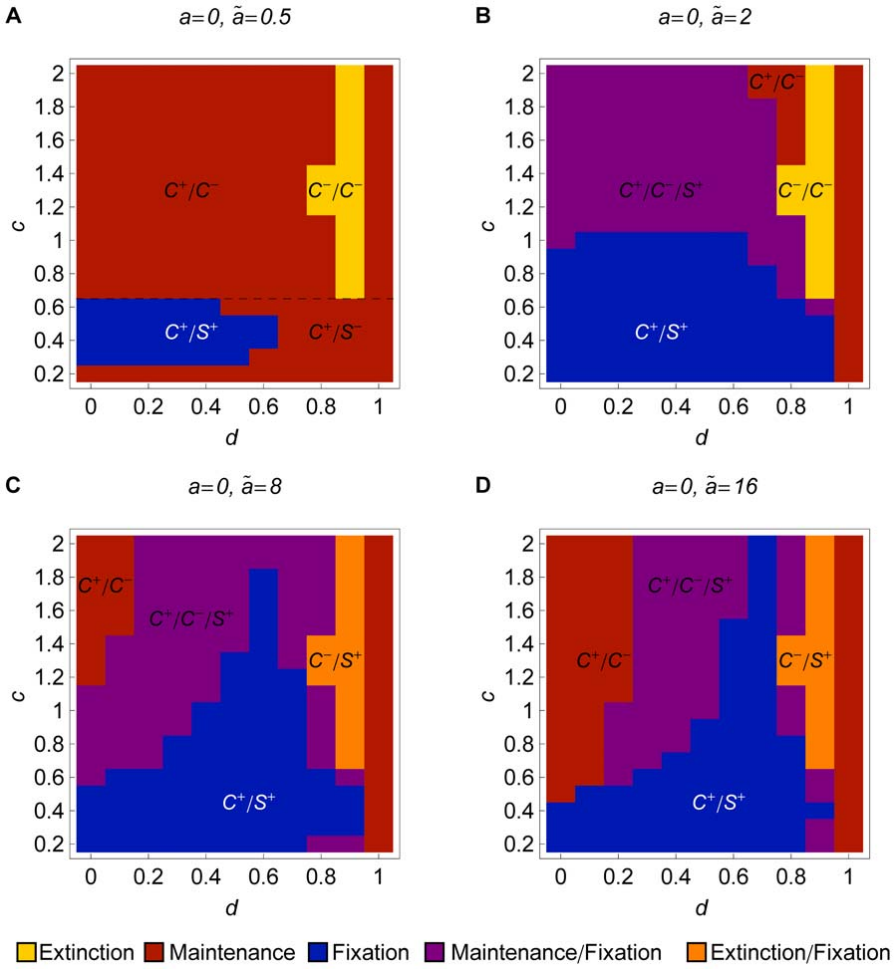
Extinction/maintenance/fixation of modifiers with different effect sizes in an initially randomly mating population. Regions of extinction, maintenance, and fixation of a modifier increasing assortment with different effects in an initially randomly mating population. The parameters 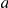, 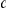, 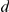, 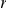, and 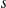 are as in Figure 3. The modifier effects are (A) 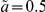, (B) 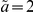, (C) 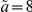, and (D) 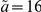. In addition to the color code, different regions are labeled 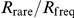 or 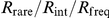, where 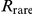, 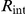, and 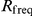 are the selection regimes that apply if the modifier is rare, at intermediate frequency, or frequent, respectively.

**Figure 5 pone-0016821-g005:**
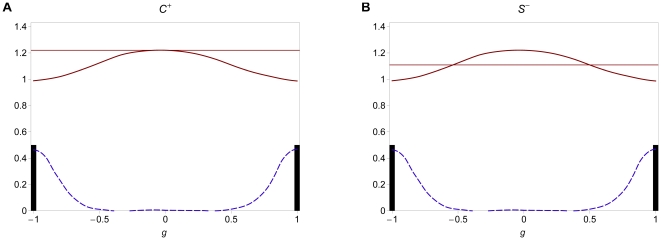
Viability and mating probability for strong assortment. Viability 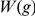 (thick solid line; cf. eq. 5), mating probability 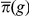 (thick dashed line) and phenotype distributions (black bars) at the fixation equilibrium if the modifier has large effect (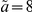). In A, there is no dominance and the modifier cannot go to fixation. In B, dominance is intermediate 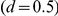 and the modifier goes to fixation if sufficiently frequent. The strength of competition is 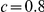 in both figures. Furthermore, 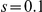 and 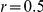. Thin straight lines show the viability (solid) and the mating probability (dashed) of heterozygotes. Equilibrium frequencies of homozygotes at the ecological locus are indicated by black bars. The equilibrium frequencies of heterozygotes are negligible and not visible.

**Figure 6:**
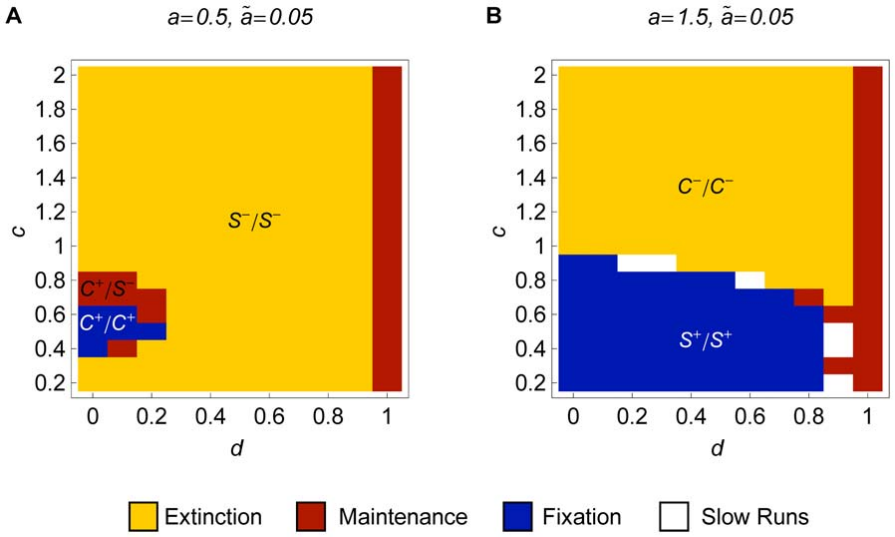
Extinction/maintenance/fixation of small-effect modifiers in an initially assortatively mating population. Regions of extinction, maintenance, and fixation of a modifier increasing assortment slightly (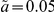). The parameters 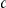, 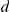, 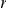, and 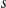 are as in Figure 4. The degree of initial assortment is (A) 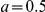 and (B) 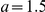.

As seen in Figure 3, higher levels of assortment are favored according to the 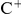 regime in almost the whole parameter space. If viability (5) is 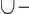
shaped and positive frequency-dependence is absent or weak (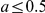), the 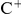 regime applies: Two niches exist at the boundary of the phenotypic range, and stabilizing sexual selection is too weak to counteract disruptive selection resulting from competition (Figure 1A). Therefore, higher levels of assortment are favored in this scenario. Dominance weakens disruptive selection at the ecological locus. Thus, this scenario is not very robust to changes in the degree of dominance. We will see, the region in which this scenario applies decreases with increasing assortment.

From Figure 3 it becomes clear that assortment cannot evolve at all only if dominance is almost complete (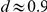) and competition is at least moderately strong (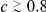). Then, (5) is 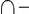
shaped and the 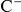 regime applies: Assortative mating and competition are strong enough to establish a niche in the middle of the phenotypic range. In addition, competition is strong relative to assortative mating, such that the net effect of selection is stabilizing. Assortative mating may induce disruptive sexual selection in this scenario (Figure 1D). However, higher levels of assortment are not favored because heterozygotes have a significantly higher viability than homozygotes. The strength of competition that is necessary to establish a niche in the middle of the phenotypic range depends crucially on the frequency of heterozygotes at the ecological locus. Since we restrict attention to at most moderate competition, i.e., 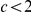, a sufficiently low frequency of heterozygotes at the ecological locus, i.e., sufficiently strong assortative mating, or sufficiently strong dominance, are necessary for the establishment of a niche in the middle of the phenotypic range. However, if the degree of dominance increases, heterozygote advantage decreases.

Whether a modifier can also go to fixation depends crucially on competition and dominance. Remember that in the quadratic model without dominance, a modifier with small effect 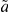 goes to fixation if competition is sufficiently strong, i.e., 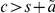. This results needs to be modified in the full model with dominance. Since dominance decreases the effect of competition, we expect the threshold value of 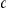 for fixation to increase with 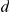. In fact, a modifier cannot go to fixation if 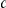 is small and 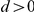 (see 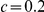 in Figure 3). If the modifier is close to fixation, the 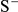 regime applies and the modifier is consequently maintained at intermediate frequency.

Generally speaking, in the 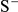 regime assortment is moderately strong and competition is comparatively weak. Heterozygotes are sufficiently common that sexual selection acts against their elimination (we call this stabilizing sexual selection). Stabilizing sexual selection outweighs disruptive selection resulting from competition. Hence, a 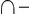
shaped phenotype distribution is optimal and higher levels of assortment are disadvantageous. Competition can be weak (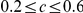, Figure 1E) or moderate (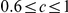, Figure 1F) in this scenario. Dominance increases the parameter region in which this scenario applies. In particular, dominance hinders heterozygotes to exploit a niche in the middle of the phenotype range (Figure 1F).

Small assortment modifiers cannot go to fixation if the degree of dominance exceeds a critical value (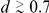). The reason is that disruptive selection is very weak for sufficiently strong dominance. If the strength of assortment increases, selection becomes stabilizing. If 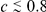, the 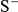 regime applies for a sufficiently frequent modifier. If 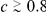, the 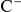 regime applies for a sufficiently frequent modifier. In both cases, a modifier will spread while rare, but cannot go to fixation.

#### 3.3.2 Size of the modifier effect

As discussed in Section 3.2, the size of the modifier effect plays a crucial role in the evolution of assortment. Assortment reduces the frequency of heterozygotes at the ecological locus. Hence, it increases the viability of individuals in the middle of the phenotypic range. Moreover, assortative mating induces sexual selection, which can be stabilizing or disruptive, depending on the strength of assortment. Finally, if assortment is very strong, selection at the modifier locus will be very inefficient because the frequency of heterozygotes at the ecological locus is strongly reduced. For a fixed set of parameters, different regimes can apply at different points in time, especially if modifier effects are large. This may result in multiple stable equilibria. An initially rare modifier with large effect can become fixed only if sufficiently strong disruptive sexual selection is established during its sweep. Figure 4 illustrates the evolutionary outcome of modifiers with different effect sizes. Note that the effect size does not affect the region in which an initially rare modifier is lost. The reasons for loss of modifiers are the same as in the case of small effects. In contrast, the fixation region depends in a nonlinear and complicated way on the modifier effect and the initial frequency of the modifier.

First, consider a modifier with effect 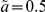 (Figure 4A). Again, multiple stable equilibria were not detected. The fixation region collapses to a narrow region in the parameter space (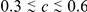 and 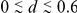). In this region, the 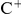 regime applies for a rare modifier, whereas the 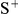 regime applies for a frequent modifier.

In the 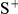 regime, assortment is sufficiently strong (compared with competition) to reduce the overall fitness of heterozygotes at the ecological locus to an extent that overall disruptive selection is established. This can be accomplished if competition is weak (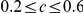, see Figure 1B) or moderate (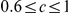, Figure 1C). Then, because their frequency is relatively low, heterozygous males pay higher costs for being rare. Consequently, an increase in assortative mating is favored. However, selection at the modifier locus is weak because of the low frequency of heterozygotes at the ecological locus. In addition, dominance decreases the difference between phenotypic values of heterozygotes and homozygotes. Therefore, selection for assortment can be very weak in this scenario.

If 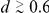 and 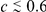, heterozygotes at the ecological locus are less fit than homozygotes for a sufficiently rare modifier (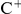). If the modifier increases in frequency the 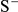 regime applies since competition in the middle of the phenotype range is reduced because of dominance and assortment. Consequently, the modifier cannot become fixed. If 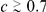, competition is strong enough to establish a niche in the middle of the phenotypic range during the spread of a modifier, i.e., the 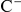 regime applies for a sufficiently frequent modifier. As a rule of thumb, modifiers with moderately large effect can only go to fixation if they manage to jump the “gap” in which the 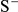 or 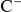 regime applies (cf. Figure 2).

If modifiers have large effect (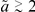), disruptive sexual selection is strong for frequent modifiers. Therefore, initially frequent modifiers go to fixation in a wide parameter range. These parameter ranges are hatched in Figures 4B–D. However, fixation was only observed if the modifier is initially at very high frequency, i.e., in the frequent-modifier scenario. Since we are primarily interested in the build up of reproductive isolation, we restrict attention to the standard and the rare-modifier scenario for the rest of the section.

If the modifier effect is moderately strong (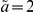; Figure 4B), the fixation region increases compared to the case 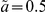. In particular, a broader range of values for 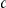 permits fixation of the modifier. The reason is that the 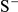 (for small 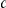), and 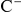 (for moderately large 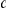) regimes are less likely to occur during the spread of modifiers with sufficiently large effect. The range for 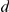 in which modifiers become fixed also increases compared to the case 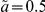. Weak disruptive selection is sufficient for invasion. This occurs if 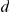 is large. If a modifier increases in frequency, strong disruptive sexual selection will be established and the modifier will go to fixation. Interestingly, intermediate dominance is most favorable for fixation of a modifier. If the level of assortment increases in (a part of) the population, a niche in the middle of the phenotypic range may be established. Dominance impedes heterozygotes to exploit such a niche (cf. Figure 1C). This means that the 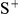 regime can be easier established if dominance is moderately strong. If dominance is strong, the mating success of homozygotes and heterozygotes at the ecological locus is almost identical. If competition is sufficiently strong, the regime 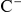 applies as the modifier rises in frequency. Consequently, an initially rare modifier does not become fixed if the degree of dominance is high and competition is at least moderately strong. This explains why intermediate dominance maximizes the size of the fixation region.

Next, we consider modifiers that lead to (almost) complete reproductive isolation if fixed. Figures 4C and D illustrate the fate of modifiers with effects 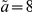 and 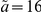, respectively. Quite surprisingly, the positive effect of dominance on the fixation of modifiers is most pronounced if modifiers have large effects. Strong assortment, which is quickly established if modifiers have large effect, leads to extremely strong disruptive sexual selection. If 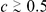, dominance is necessary for fixation of the modifier. In the absence of dominance and if 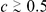, the reduced mating success of heterozygotes is compensated by the emergence of a niche in the middle of the phenotype range as the modifier becomes sufficiently frequent (Figure 5A). Consequently, an initially rare modifier will not spread to fixation. The presence of dominance does not change the strength of sexual selection unless it is sufficiently strong (Figure 5B). The “valley” of low mating probabilities in the middle of the phenotypic range becomes deeper and flatter with increasing assortment. Dominance has almost no effect on the strength of disruptive sexual selection as long as the phenotypic value of heterozygotes at the ecological locus stays in this valley. In contrast, if dominance increases, the viability of heterozygotes decreases strongly (Figure 5B). This explains why the optimal degree of dominance increases with increasing modifier effect.

#### 3.3.3 Dependence on the initial level of assortment

Figure 6 illustrates the evolutionary outcome of modifiers with small effect for various initial degrees of assortment. Multiple stable equilibria were not detected. Even a small amount of initial assortment leads to a substantial change of the region in which modifiers are maintained. The maintenance region shrinks with increasing initial assortment and approaches its minimum at 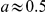 (see Figure 6A). If assortment is weak (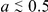), sexual selection is stabilizing. Thus, the 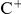 region decreases with increasing assortment. If competition is weak (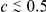), stabilizing sexual selection outweighs disruptive selection at the ecological locus and the 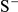 regime applies. Furthermore, dominance decreases the effect of competition. Therefore, if competition is weak the 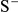 region is established for weaker assortment. For strong competition (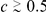), a niche in the middle of the phenotype spectrum can be established if the frequency of heterozygotes is reduced. Thus, the 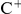 region is replaced by the 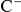 region if initial assortment increases.

The fixation and maintenance regions increase with increasing initial degree of assortment if 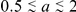. Then, disruptive sexual selection can be established as long as 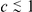. Dominance slightly decreases the region in which a modifier is maintained or goes to fixation. However, the effect of dominance is less pronounced compared with the case of weak initial assortment. For moderately strong initial assortment, evolution can be very slow such that slow runs are observed. For strong assortment (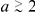) only slow runs are observed (data not shown). This is consistent with our results about invasion fitness. We conclude that establishment of high levels of assortment via a series of invasion and fixation of modifiers with small effect seems unlikely.

### 3.4 Evolution of assortative mating

The build-up of reproductive isolation via allele substitutions of initially rare modifiers with small effects faces several problems. Positive frequency-dependence due to an intermediate level of assortment can lead to overall stabilizing selection because it outweighs disruptive selection resulting from competition. On the other hand, for weak or moderate assortment, and sufficiently strong competition, a niche in the middle of the phenotype range appears if heterozygotes become sufficiently rare. Finally, for high levels of assortment, a severely reduced frequency of heterozygotes can neutralize selection at the modifier locus.

**Figure 7:**
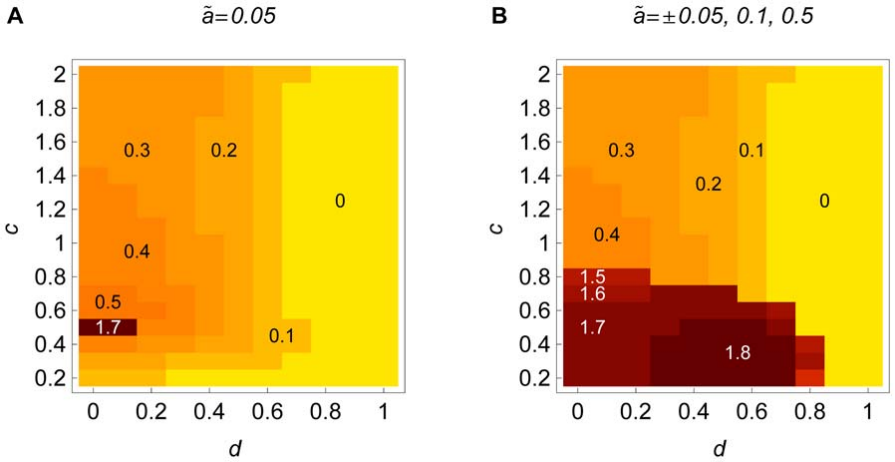
Evolutionary stable degrees of assortment. Evolutionary stable degrees of assortment that can evolve via allele substitutions of initially rare modifiers if modifiers have small positive effect (A), or various positive or negative effects (B). The parameters 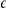, 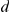, 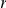, and 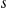 are as in Figure 4. The numbers in the differently shaded regions indicate the maximum degree of assortment that can evolve (starting from random mating).

Our approach allows us to construct sequences of invasion and fixation of modifiers with different effects. If we consider only initially rare modifiers with small effect, we obtain an estimate for the degree of assortment that can evolve by small steps. Figure 7A shows that only low levels of assortment can evolve, except for a small region of moderate competition and very weak dominance. Furthermore, assortment does not evolve above a moderate level (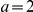).

In Figure 7B we consider modifiers of slightly larger effect and also allow modifiers with negative effect (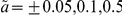). This gives us an estimate for the evolutionary stable degree of assortment. If modifier effects are small, but sufficiently large to jump over the gap described in Section 3.3.3, the region in which moderate assortment evolves increases substantially. However, strong levels of assortment, which are necessary for speciation, cannot evolve.

Thus, we conclude that evolution of assortment is most likely if modifier effects are large, so that complete reproductive isolation can be established in a single step. However, a moderately strong degree of dominance is favorable for the evolution of strong reproductive isolation and hence also for sympatric speciation.

### 3.5 Rate of evolution

**Figure 8:**
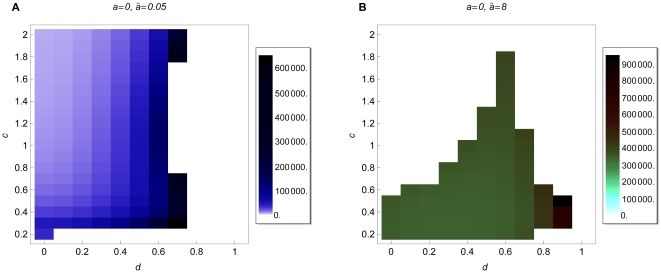
Mean fixation time. Mean fixation time of an initially rare modifier with small (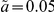, A), or large (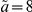, B) effect. For each parameter combination the mean fixation time was calculated as the average fixation time of the 10 respective runs with different initial frequencies. The parameters 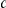, 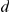, 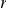, and 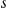 are as in Figure 4. Note that we used different scales in the figures.

It is not only relevant whether modifiers become fixed, but also whether this happens within a biologically meaningful time. Therefore, for a fixed parameter combination, we recorded the mean fixation time of a modifier (over all initial conditions). Figure 8 shows the mean fixation time of initially rare modifiers in an initially randomly mating population. If the modifier effect is small (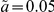, Figure 8A), the 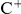 regime applies during the spread of a modifier, and dominance mainly weakens disruptive selection at the ecological locus. If the modifier effect is large (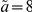, Figure 8B), the time until fixation is much longer compared to modifiers with small effect. Initially, while the 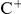 regime applies, selection for modifiers with large effect is stronger than for modifiers with small effect. However, the frequency of heterozygotes is reduced very quickly and then the 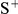 regime applies until fixation. As discussed above, selection is very weak in the 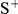 regime. Therefore, the time until fixation increases for modifiers with larger effects. Similarly, the time until fixation increases with increasing initial assortment (data not shown).

### 3.6 Speciation

The evolution of sufficiently high levels of reproductive isolation can lead to speciation. By speciation we mean that the population is split into two different phenotypic clusters with hardly any gene flow between the clusters. We shall say there occurs speciation if the probability that two individuals with genotypes 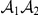 and 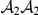 at the ecological locus mate is less than the threshold 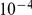.

The critical threshold for the strength of assortment that is necessary for speciation depends on the strength of competition and dominance. One should mention that indirect selection is already very weak for 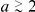. Thus, the occurrence of speciation may depend critically on the threshold values of the mating probability. Smaller thresholds require larger 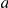 for speciation. In our case, the critical level of assortment necessary for speciation is 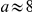.

**Figure 9:**
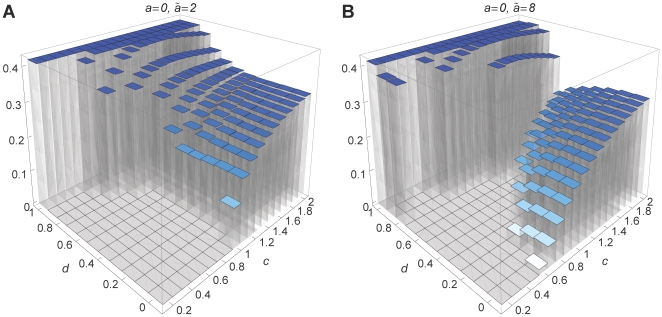
Equilibrium frequency of heterozygotes. Frequency of heterozygotes at the ecological locus at equilibrium. The parameters 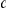, 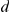, 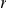, and 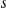 are as in Figure 4. Moreover, 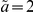 in A, 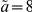 in B, and 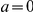 in both figures.

Our results show that establishment of sufficiently high degrees of assortment for the occurrence of speciation is unlikely if modifiers have small effects. If the population mates initially randomly and modifiers have a sufficiently large effect (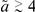), speciation occurs in the parameter range in which modifiers become fixed. In the regions, in which modifiers are maintained at intermediate frequency, speciation could occur as well, at least theoretically. Our analysis shows, that the region in which speciation occurs coincides exactly with the fixation regions of modifiers with sufficiently large effect. This suggests that our results are robust with respect to changes in the threshold value in our definition of speciation. In fact, the equilibrium frequency of heterozygotes at the ecological locus is quite high in the maintenance regions. Figure 9 shows the frequency of heterozygotes at equilibrium for a modifier with effect 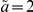 (A) and 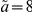 (B) in an initially randomly mating population. We conclude that fixation of modifiers with sufficiently large effect is necessary for speciation.

## Discussion

Intraspecific competition, or, more generally, negative frequency-dependent selection, is a commonly used ecological setup to model the evolution of assortment and sympatric speciation (e.g., [10], [12], [13], [29], [38]). The African finch *Pyrenestes Ostrinus*, an often-cited justification for this ecological setup, however, did not evolve assortment [23], [24], but avoids unfit heterozygotes because one morph is completely dominant. Assortative mating and dominance are commonly considered as alternative evolutionary responses to avoid heterozygous disadvantage (e.g., [30]). However, the importance of the interactions of assortment and dominance is emphasized in [34].

Here, we studied the evolution of assortative mating under intraspecific competition in the presence of dominance. In our model, a single diallelic (ecological) locus has a major effect on a quantitative trait under a mixture of stabilizing selection, intraspecific competition, and density regulation. The trait expresses an arbitrary degree of intermediate dominance. An additional diallelic (modifier) locus determines the strength of assortative mating with respect to the ecological trait (‘magic trait’, cf. [15]). Assortative mating follows the model of Matessi et al. [12], which is based on the original formulation by Gavrilets and Boake [35]: choosiness is expressed only in females, who pick their mates based on similarities in their trait values. Although our model ignores direct costs for choosy females, assortative mating induces sexual selection, which may be stabilizing or disruptive, depending on the strength of assortment.

In our model, negative frequency dependence (caused by intraspecific competition) favors sufficiently different and rare types. This is opposed by positive frequency dependence (caused by assortative mating) selecting for similar and common types. The amount of competition and sexual selection experienced by the individuals changes as assortment evolves because the frequency of heterozygotes (at the ecological locus) changes. Hence, as assortment increases, selection becomes less efficiently transmitted from the ecological to the modifier locus. Since, for given parameters, it is not straightforward which selective components contribute most to the final evolutionary outcome, we identified four different selection regimes (see Results) that are helpful in interpreting our results.

Heterozygotes are common if assortment is weak. Then, sufficiently strong competition leads to disruptive selection, i.e., selection for higher levels of assortment (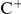 regime, Figure 1A). If competition is too weak, stabilizing (sexual) selection dominates and assortment cannot evolve (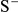 regime, Figure 1E and F). Strong assortment induces disruptive sexual selection because heterozygotes are deleterious when rare. Therefore, if assortment is sufficiently strong relative to competition, even stronger assortment can evolve (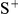 regime, Figure 1B and C). However, the disadvantage of heterozygotes can be compensated by very strong competition to the extent that assortment cannot evolve (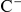 regime, Figure 1D).

We derived simple invasion and fixation conditions under the assumptions of weak selection and/or weak assortative mating. For initially weak assortment and in the absence of dominance, higher levels of assortment can evolve whenever competition is sufficiently strong (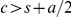; 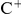 regime). Modifiers with small effect do not necessarily go to fixation if they can invade (because the 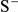 regime may apply if modifiers become frequent). In contrast, modifiers with large effect become fixed if sufficiently frequent (because the 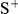 regime applies if modifiers of large effect become frequent). Thus, strong assortment evolves easier if modifiers have large effects. In a randomly mating population with no, weak or almost complete dominance, assortment can evolve if 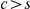. Hence, dominance has no significant effect on the initial evolution of assortment if it starts from random mating. If assortment is complete, modifiers decreasing assortment by an arbitrary amount cannot invade as long as dominance is incomplete or the mating probability between homozygotes (at the ecological locus) becomes positive.

The complexity of the model prohibits further analytical investigations. Thus, we pursued a thorough numerical approach to study arbitrarily strong assortment and competition, and different modifier effect sizes. We focused on parameter combinations that lead to disruptive selection under random mating. Hence, an initial increase of assortment occurred almost in the whole parameter space (cf. Figure 3). However, the modifier's fixation region depends strongly on its initial frequency, the size of its effect, and the degree of dominance.

For small modifiers (Figures 2, 3, 6, 7A) complete assortment can evolve only if competition is moderately strong and dominance is weak (see Figures 2 and 7A). If competition is weak, only partial reproductive isolation can evolve because stabilizing sexual selection neutralizes disruptive selection due to competition (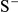) (cf. [12], [13], [31]). For sufficiently strong competition, intermediate phenotypes become advantageous as assortment increases (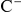) (cf. [13]). Noteworthy, the 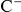 regime does not exist in the quadratic model. Therefore, it was not detected in [12]. In general, dominance decreases the parameter range in which assortment can evolve because the regimes 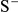 or 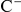 are easier established if there is dominance. The evolutionary stable degree of assortment that can be achieved by a series of modifiers decreases significantly with increasing dominance (see Figure 7A). This complements the findings of Durinx and van Dooren [30], who claimed that dominance hinders the evolution of assortment.

Disruptive sexual selection can be established readily during the spread of large modifiers. An initially rare, sufficiently large modifier can jump across the gap in which either the S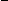 or C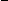 regime applies (cf. Figure 2). Thus, in some parameter regions only sufficiently large modifiers can become fixed (cf. Figure 3 and 4). In particular, dominance supports the evolution of reproductive isolation if modifiers have sufficiently large effect. The reason is that small degrees of dominance have little effect on the strength of disruptive sexual selection if assortment is sufficiently strong, but the viability disadvantage of heterozygotes vanishes as dominance increases (see Figure 5). This effect is reversed for very strong dominance. Hence, intermediate dominance is optimal for the evolution of assortment in large steps. Moreover, as assortment increases, higher levels of dominance become necessary to compensate heterozygote disadvantage resulting from sexual selection. Therefore, the optimal degree of dominance increases with increasing modifier effect. It should be mentioned that in a wide parameter range (hatched area in Figure 4) assortment cannot be decreased by rare modifiers (of small or large effect).

We also studied fixation times of initially rare modifiers. The evolution of assortment is very slow if sexual selection is the driving force for fixation (see Figure 8B). By no means can invasion fitness be used as a proxy for fixation time. Although the initial strength of selection increases with increasing modifier effect, fixation of large modifiers usually takes longer than fixation of small modifiers (cf. Figures 8A and B). Furthermore, the fixation time of large modifiers is minimized for intermediate dominance.

Finally, we briefly studied the occurrence of speciation in our model. Modifiers with large effect are much more likely to establish strong reproductive isolation, a prerequisite for speciation. For such modifiers, our results suggest that intermediate dominance is most supportive for sympatric speciation. In general, the build-up of strong reproductive isolation is rather slow. The reason is that selection at the modifier locus is very weak if heterozygotes at the ecological locus become rare. In a natural population, evolution of assortment might stop at some intermediate level. Only if sufficiently strong assortment evolves by a single allele substitution, the occurrence of speciation seems likely.

Our present results combined with those in [34] allow us to draw conclusions about the simultaneous evolution of dominance and assortment. In [34] the same ecological model is studied, but the level of assortment is a fixed parameter and the degree of dominance evolves. As shown there, the evolution of dominance is impeded by small degrees of assortment but enhanced by intermediate degrees. In particular, time to fixation is minimized for modifiers inducing complete dominance and intermediate assortment. Together with previous results in [34], [37], our results show that fixation times of dominance modifiers are usually shorter than of assortment modifiers. Hence, we conclude that complete dominance is often the more likely evolutionary outcome. However, mutation rates and mutational step sizes play decisive roles in the simultaneous evolution of dominance and assortment. We expect that neither complete dominance, nor complete assortment will evolve unless one of them evolves very quickly. This coincides with the fact that dominance can support the evolution of reproductive isolation via large modifiers (which are initially rare), but hinders the evolution of intermediate levels of assortment in small steps (see Figure 7A).

Note that our model does not incorporate (direct) costs for choosiness. Although weak costs for choosiness do not necessarily prohibit the evolution of strong assortative mating (cf. [13], [31], [39]) it becomes less likely. This coincides with our conclusion that complete dominance is more likely to evolve than complete assortative mating.

A crucial assumption in our study is that alleles have symmetric effects, which implies that homozygotes at the ecological locus have symmetric phenotypes. This assumption might seem artificial since dominance breaks down any symmetry in the model anyway. For asymmetric effects it is likely that our results change. Namely, dominance will outbalance the asymmetry of the effects, and create a situation similar to one with symmetric effects and a different level of dominance. Hence, we expect that the level of dominance which is optimal for the evolution of stronger assortment shifts. In particular, we suggest that assortment shall evolve easier if the allele with the smaller (absolute) effect expresses some degree of dominance (the opposite is true for dominance towards the allele with the larger effect). Especially, we expect that assortment can evolve best for degrees of dominance that are higher than in the symmetric case. The reason is that assortative mating induces positive frequency-dependent selection and counteracts intraspecific competition. If the allele with the smaller effect expresses dominance, phenotypes near the optimum of stabilizing selection experience strong intraspecific competition, which can be compensated by higher levels of assortative mating. However, our study already revealed the complex interactions of dominance and assortative mating for symmetric allelic effects. These interactions will become even more complex if the symmetry assumption is relaxed. In particular, density-dependence might be profoundly influential for asymmetric effects, which might disprove our above reasoning.

Our study differs from previous work on the evolution of assortment because we explicitly studied the effect of dominance and considered the global dynamics. We studied a large part of the parameter space, including intermediate levels of assortment and large modifier effects, and detected previously unobserved phenomena. Moreover, we can draw conclusions on the simultaneous evolution of assortative mating and dominance.

Durinx and van Dooren [30] studied the evolution of dominance and assortative mating using an adaptive-dynamics approach. They compared the invasion fitness of dominance and assortment modifiers of small effect, and concluded that dominance and assortment are mutually exclusive alternatives, and the occurrence of one decreases the likelihood of the other. Our results yield a more complete picture. Dominance hinders the evolution of assortment if modifier effects are small, but promotes it if they are large. A detailed discussion of the differences between the present approach and the one used in [30] can be found in [34].

The importance of modifiers of large effect, which may overcome the gap in which either the 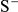, or the 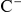 regime applies, was also pointed out in [13]. There, the evolution of assortative mating in a two-locus two-allele version of the model used by Dieckmann and Doebeli [10] was explored. Notably, they used a different ecological model [16], assumed no dominance, and considered several forms of competition. In the absence of dominance, their results are similar to ours. For large modifier effects, their results rely on individual-based simulations and suggest that complete assortment evolves within reasonable time if mutations at the modifier locus are sufficiently large and frequent. Our model, however, suggests that the evolution of strong assortative mating takes very long. Apparently, small population sizes and high mutation rates strongly facilitate the evolution of complete reproductive isolation (see also [10], [40], [41]).

Otto et al. [31] investigated the evolution of assortment in a more general two-locus two-allele model, based on a local stability analysis and a quasi-linkage equilibrium (QLE) approach. They studied different forms of assortment and found simple conditions for the evolution of assortative mating. In the absence of costs, higher levels of assortative mating are favored when homozygotes are, on average, fitter than heterozygotes. However, their derivations often required absence of dominance or weak selection, and the QLE assumption might be problematic for strong assortment. Interestingly, they found that dominance can promote the evolution of assortment under directional selection, i.e., assortment can evolve during a selective sweep of a partially recessive, beneficial mutation. Moreover, assortative mating evolves easier without sexual selection, provided viability selection is disruptive (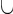-shaped). However, in models of intraspecific competition, rare heterozygotes can be at a fitness maximum, which would stop the evolution of assortment in the absence of sexual selection. In our model, dominance supports the evolution of assortment only if there is disruptive sexual selection.

As in [12], [13], [30], [31] we assumed that a single diallelic locus determines the trait value. Although the equilibrium structures are largely consistent with those in multi-locus models [9], [10], [42], [43], in the latter more than two reproductively isolated species can evolve [36], [42]. A recent study of a multilocus version of the model studied in [13] performed by Rettelbach et al. [44] shows that the genetic architecture of the ecological trait hardly influences the parameter range in which two reproductively isolated species can evolve. Since, in multilocus competition models, disruptive selection often concentrates all genetic variation at a single locus [45], [46], our result should extend to such cases. However, some caution is necessary because the maintenance of multilocus polymorphism depends highly on genetic constraints, (cf. [47]).

A recent study of a multilocus system found that the evolution of assortment requires underdominance or epistasis at the fitness level [32]. Hence, intermediate dominance at the trait level may have important consequences in multilocus models for the evolution of assortment and deserves further attention. Noteworthy, in [32], intermediate degrees of assortment were not evolutionary stable, which disagrees with our results and those in [12], [13], [31]. Results on multilocus models (e.g., [9], [10], [42]–[44]) suggest that the disagreement is not a consequence of the genetic architecture, but is due to the different assumptions about selection.

All this suggests that our results are robust with respect to variations in the specific model of intraspecific competition, but highly dependent on the assumptions about assortative mating. Our results should continue to hold as long as assortative mating induces positive frequency-dependent selection. Predicting the robustness of our results to changes in the genetic architecture seems more difficult. We expect our results to hold in multilocus models if intraspecific competition causes negative frequency-dependent selection.

We showed that dominance and assortment are not necessarily exclusive alternative responses to disruptive selection. However, unless modifiers have large effects, already quite low degrees of dominance severely limit the potential for the evolution of female choosiness.

Our results suggest that dominance is the more likely evolutionary response to intraspecific competition. Furthermore, we emphasized the importance of studying global dynamics and the limitations of invasion fitness approaches. However, the evolution of assortment or dominance is not the only possible response to disruptive selection [48]. Other responses include the evolution of sexual dimorphism [49], niche width [50], and bet hedging [51]. The co-evolution of genetic architecture, individual specialization, and assortative mating is a fascinating area of research that still harbors many challenges for future studies.

## Supporting Information

Appendix S1 **Appendix.**
(PDF)
