# Supplementary material for: Evolution of Assortative Mating in a Population Expressing Dominance
Source: PLoS One. 2011 Apr 1;6(4):e16821. doi: 10.1371/journal.pone.0016821 (PMC3069974; doi:10.1371/journal.pone.0016821)
Supplement: Appendix S1 — Appendix. (PDF) [file pone.0016821.s001.pdf]

Appendix S1: Evolution of assortative mating in a population expressing dominance

Kristan A. Schneider[1,*], Stephan Peischl[1]
1 Department of Mathematics, University of Vienna, Vienna, Austria
∗ E-mail: kristan.schneider@univie.ac.at

S1 Invasion and fixation of assortment modifiers

Here, we derive invasion and fixation conditions for modifiers inducing stronger or weaker assortment. We label the genotypic values and the genotype frequencies according to Table S1.

In all cases, we assume that fitness is given by equation (8) in the main text and that population size is constant and close to demographic equilibrium. To derive invasion conditions we can neglect matings between individuals carrying a modifier allele, i.e., $\mathcal{O}(p_i p_j)$ terms ($i,j \in \{4,\dots,10\}$). Moreover, the genetic composition of the population is adequately described by the vector $(p_1,p_2,p_3,p_4,p_5,p_6,p_7)^T$. It is easily verified that the linearized recursion matrix of the vector $(p_4,p_5,p_6,p_7)^T$ is

$$U = \frac{1}{\overline{\overline{W}}}\begin{pmatrix} W_1\alpha_1 & W_5\beta_1 r & W_5\beta_2(1-r) & 0 \\ 0 & W_5\beta_1(1-r) & W_5\beta_2 r & W_3\gamma_1 \\ W_1\alpha_2 & W_5\beta_3 r & W_5\beta_4(1-r) & 0 \\ 0 & W_5\beta_3(1-r) & W_5\beta_4 r & W_3\gamma_2 \end{pmatrix}, \tag{S1}$$

where

$$\begin{aligned}
\alpha_1 &= \frac{1}{2}p_1^*(Q_{14}^*+Q_{41}^*)+\frac{1}{4}p_2^*(Q_{24}^*+Q_{42}^*), && \text{(S2a)}\\
\alpha_2 &= \frac{1}{2}p_3^*(Q_{34}^*+Q_{43}^*)+\frac{1}{4}p_2^*(Q_{24}^*+Q_{42}^*), && \text{(S2b)}\\
\beta_1 &= \frac{1}{2}p_1^*(Q_{15}^*+Q_{51}^*)+\frac{1}{4}p_2^*(Q_{25}^*+Q_{52}^*), && \text{(S2c)}\\
\beta_2 &= \frac{1}{2}p_1^*(Q_{16}^*+Q_{61}^*)+\frac{1}{4}p_2^*(Q_{26}^*+Q_{62}^*), && \text{(S2d)}\\
\beta_3 &= \frac{1}{2}p_3^*(Q_{35}^*+Q_{53}^*)+\frac{1}{4}p_2^*(Q_{25}^*+Q_{52}^*), && \text{(S2e)}\\
\beta_4 &= \frac{1}{2}p_3^*(Q_{36}^*+Q_{63}^*)+\frac{1}{4}p_2^*(Q_{26}^*+Q_{62}^*), && \text{(S2f)}\\
\gamma_1 &= \frac{1}{2}p_1^*(Q_{17}^*+Q_{71}^*)+\frac{1}{4}p_2^*(Q_{27}^*+Q_{72}^*), && \text{(S2g)}\\
\gamma_2 &= \frac{1}{2}p_3^*(Q_{37}^*+Q_{73}^*)+\frac{1}{4}p_2^*(Q_{27}^*+Q_{72}^*). && \text{(S2h)}
\end{aligned}$$

Recall that an asterisk indicates that genotype frequencies after selection are used, and that r denotes the recombination rate between ecological locus and modifier locus. The genotype frequencies of individuals carrying exactly one copy of the modifier in the next generation are then given by $U \cdot (p_4,p_5,p_6,p_7)^T$. If the leading eigenvalue of U is larger than 1 in modulus, the modifier will spread, otherwise it will vanish. Similarly, one can determine whether a sufficiently frequent modifier will rise to fixation or not. Clearly, for $\tilde{a}=0$ the modifier allele is selectively neutral and the leading eigenvalue λ of U is 1.

For a modifier with small effect, i.e., $\tilde{a}-a$ is small, the leading eigenvalue of U can be written as

$$\lambda = 1 + \tilde{a}\phi(a,s,c,d) + O(\tilde{a}^2)\,. \tag{S3}$$

The sign of ϕ determines whether a modifier can invade or not. A rare modifier spreads if and only if $\phi > 0$.

Note that it follows from (S3) that a modifier decreasing assortment invades if and only if $\phi < 0$.

S1.1 No dominance, small modifier effect

We start by observing that the case of no dominance allows a few simplifications of the matrix U. We restrict our attention to symmetric equilibria, i.e., $\hat{p}_1 = \hat{p}_3$, since it seems infeasible to calculate polymorphic asymmetric equilibria explicitly. However, this assumption is justified. If the modifier locus is fixed and codes for random mating, the symmetric equilibrium $\hat{p}_1 = \hat{p}_3 = 1/4$ is globally stable, as we shall argue below. If the modifier locus is fixed and codes for complete assortment, the symmetric equilibrium $\hat{p}_1 = \hat{p}_3 = 1/2$ is the only locally stable equilibrium (see below). For all other cases, we assume that assortment is either weak or almost complete such that stable equilibria with large regions of attraction exist sufficiently close to the symmetric equilibria.

At a symmetric equilibrium, the matrix U simplifies to

$$\frac{1}{\overline{W}}\begin{pmatrix} W_1\alpha_1 & W_5\beta_1 r & W_5\beta_1(1-r) & 0 \\ 0 & W_5\beta_1(1-r) & W_5\beta_1 r & W_3\gamma_1 \\ W_1\gamma_1 & W_5\beta_1 r & W_5\beta_1(1-r) & 0 \\ 0 & W_5\beta_1(1-r) & W_5\beta_1 r & W_3\alpha_1 \end{pmatrix}, \tag{S4}$$

since

$$\alpha_1 = \gamma_2, \quad \alpha_2 = \gamma_1, \quad \beta_1 = \beta_2 = \beta_3 = \beta_4$$

holds at a symmetric equilibrium with no dominance. Furthermore, $W_1 = W_3$. Then, the characteristic polynomial of $\overline{W}U$ is

$$\begin{aligned} P(x) =& \Big(x^2 - x(\alpha_1 W_1 + \beta_1(1-2r)W_5) + \beta_1(\alpha_1 - \gamma_1)W_1W_5\Big) \\ &\times \Big(x^2 - x(\alpha_1 W_1 + \beta_1 W_5) + \beta_1(\alpha_1 - \gamma_1)W_1W_5\Big). \end{aligned} \tag{S5}$$

As shown in [1], the leading eigenvalue of U is the larger of the two solutions of

$$x^2 - x(\alpha_1 W_1 + \beta_1 W_5) + \beta_1(\alpha_1 - \gamma_1)W_1W_5 = 0 \tag{S6}$$

divided by $\overline{W}$. Since U is irreducible, the leading eigenvalue is positive and simple. For symmetric equilibria, i.e., $\hat{p}_1 = \hat{p}_3$, it is also shown in [1] that the leading eigenvalue is larger than one if and only if

$$\Delta = \frac{W_1}{\overline{W}}\Big(1 - \frac{1}{2}(Q_{12}^* - Q_{21}^*)p_2\Big) > 1. \tag{S7}$$

To derive useful invasion conditions from (S7) one needs to know the gene frequencies at equilibrium.

S1.1.1 Weak initial assortment

Assume that the wild-type allele is fixed at the modifier locus. If the wild-type allele codes for random mating, we obtain the following equilibrium

$$\hat{p}_1 = \hat{p}_3 = \frac{1}{4}, \text{ and } \hat{p}_2 = \frac{1}{2}. \tag{S8}$$

This equilibrium is the only polymorphic equilibrium, and it is locally asymptotically stable, whereas the monomorphic equilibria are unstable [2]. Since the case of random mating falls into the class of

models studied in [3] with non-negative interaction coefficients, it follows from there that the polymorphic equilibrium is globally asymptotically stable.

Now, consider a small degree of initial assortment a. If $a \ll 1$, we obtain the following equilibrium

$$\hat{p}_1 = \hat{p}_3 = \frac{1}{4} + \frac{a\,(2-2s+3c)^2}{8\,(2-s+2c)^2} + \mathcal{O}(a^2), \text{ and } \hat{p}_2 = \frac{1}{2} - \frac{a\,(2-2s+3c)^2}{4\,(2-s+2c)^2} + \mathcal{O}(a^2)\,. \tag{S9}$$

More precisely, there necessarily exists an asymptotically stable equilibrium sufficiently near (S9).

Substitution of (S9) into (S7) yields

$$\Delta = 1 + \frac{a-2(c-s)}{4} + \mathcal{O}(a^2) + \mathcal{O}(s^2) + \mathcal{O}(as). \tag{S10}$$

Thus, under the assumption of weak selection and weak assortment the invasion condition becomes

$$c > s + \frac{a}{2}. \tag{S11}$$

This result includes the case of random mating for $a = 0$. Notably, in this case the leading eigenvalue can be calculated directly to be

$$\lambda = 1 + (\tilde{a}-a)\frac{2(c-s)-a}{16} + \mathcal{O}\big((\tilde{a}-a)^2\big). \tag{S12}$$

Moreover, we infer from (S12) that a sufficiently frequent modifier with small effect $\tilde{a}$ goes to fixation in a population in which the wild type codes for random mating if and only if $c < s + \tilde{a}$.

S1.1.2 Strong initial assortment

We set $\varepsilon = \exp(-a)$ and assume $\varepsilon \approx 0$, so that terms of order $\mathcal{O}(\varepsilon^5)$ can be neglected. Up to fourth order in ε, the symmetric equilibrium with the modifier locus fixed for the wild-type allele is

$$\hat{p}_1 = \hat{p}_3 = \frac{1}{2} - \varepsilon^4\frac{1+2c-s}{1+3c-2s} + \mathcal{O}(\varepsilon^5), \quad \text{and} \quad \hat{p}_2 = 2\varepsilon^4\frac{1+2c-s}{1+3c-2s} + \mathcal{O}(\varepsilon^5). \tag{S13}$$

More precisely, for a fixed modifier locus there necessarily exists an asymptotically stable equilibrium sufficiently near (S13). This follows because the symmetric equilibrium is globally stable under complete assortment, as we will show in Section S1.5.

The invasion criterion (S7) simplifies to

$$\varepsilon^4\frac{1+4c-3s}{1+3c-2s} + \mathcal{O}(\varepsilon^5) > 0, \tag{S14}$$

which is always satisfied since $a, c \in [0, 1/4]$.

S1.2 No dominance, large modifier effects

S1.2.1 Invasion

Assume no dominance and a population fixed for the wild-type allele at the modifier locus. The wild-type allele codes for random mating and the population is at equilibrium (S8). Moreover, we assume an a little more general function for the mating probabilities (9). Namely, if genotype g carries exactly one copy of the modifier, we set

$$\pi(g,h) = \begin{cases} 1 & if Z_g = Z_h \\ k & if |Z_g - Z_h| = 1 \\ K & if |Z_g - Z_h| = 2, \end{cases} \tag{S15}$$

where $0 \leq k, K < 1$. If $\pi(g,h)$ is given by (9), we have $k = \exp(-\tilde{a})$ and $K = k^2$. The leading eigenvalue of U, i.e, the larger of the two solutions of (S6), is

$$\lambda = 1 + \frac{(1-K)(c-s)}{2(3+5K+8k)} + \mathcal{O}(s^2). \tag{S16}$$

Thus, a modifier increasing assortment can invade a randomly mating population if and only if $c > s$.

Similarly, in an initially weakly assortatively mating population at a symmetric equilibrium close to (S8), the leading eigenvalue of U is

$$\lambda = 1 + \frac{(1-K)[2(c-s)-a]}{4(3+5K+8k)} + \mathcal{O}(a^2) + \mathcal{O}(s^2). \tag{S17}$$

Consequently, a modifier increasing assortment can invade a population that mates weakly assortatively if and only if $c > s + a/2$. Note, that for the limit, we assumed that $a \ll k, K$, i.e., that the degree of assortative mating caused by the modifier is large compared to the initial degree of assortment.

S1.2.2 Fixation

We consider a modifier with large effect $\tilde{a}$, i.e., such that $\varepsilon^2 \ll 1$, where $\varepsilon := \exp(-\tilde{a}) \ll 1$. The wild-type allele at the modifier locus codes for random mating. If the modifier is fixed, the equilibrium gene frequencies at equilibrium are given by (S13). The leading eigenvalue of U at (S13) is

$$\lambda = 1 - \varepsilon^4 \frac{1+3c-2s}{3+7c-4s} + \mathcal{O}\left(\varepsilon^6\right). \tag{S18}$$

Because $a, s \in [0, 1/4]$, $\lambda < 1$ and hence the modifier goes to fixation if it is sufficiently frequent.

S1.3 Weak dominance, random mating

The above described invasion criterion is valid only in the absence of dominance. The reason is that the symmetry of the model without dominance is crucial in the derivation of (S9). Thus, we have to derive the leading eigenvalue directly as a Taylor Series in $(\tilde{a} - a)$. Assume that the population is fixed for the wild-type allele at the modifier locus and that the wild type codes for random mating.

As shown in [2] only one polymorphic equilibrium (with fixed modifier locus) exists. This equilibrium is locally stable, whereas the monomorphic equilibria are unstable. As in Section S1.1.1 it follows from [3] that the polymorphic equilibrium is globally stable.

To calculate the globally stable equilibrium with monomorphic modifier locus coding for random mating, we assume dominance to be sufficiently weak to ignore terms of order $\mathcal{O}(d^3)$. We obtain

$$\begin{aligned} \hat{p}_1 &= \frac{(c+c\,d+s)^2}{4\,(c+s)^2} + \mathcal{O}(d^3), \\ \hat{p}_2 &= \frac{1}{2} - \frac{(c^2 d^2)}{2(c+s)^2} + \mathcal{O}(d^3), \\ \hat{p}_3 &= \frac{(c-c\,d+s)^2}{4\,(c+s)^2} + \mathcal{O}(d^3). \end{aligned} \tag{S19}$$

Consider the characteristic polynomial P of U at the equilibrium (S19). Clearly, $P(1) = 0$ for $\tilde{a} = 0$. Thus, if $\tilde{a} \neq 0$, the leading eigenvalue of U can necessarily be written as

$$\lambda = 1 + \tilde{a}\phi + \mathcal{O}(\tilde{a}^2) \tag{S20}$$

as $\tilde{a} \to 0$ for some ϕ which is independent of $\tilde{a}$. By neglecting terms of order $\mathcal{O}(\tilde{a}^2)$, the Taylor expansion of P at $\hat{p}$ leads to

$$\phi = \frac{\left(1 - 2\,d^2\right)\,(c-s)}{8} + \mathcal{O}(d^3) + \mathcal{O}(s^2)\,. \tag{S21}$$

The strength of selection for assortment modifiers consequently decreases under weak dominance. However, the invasion condition is not affected by a small degree of dominance.

S1.4 Strong dominance, random mating

We set $\delta = (1-d)$. By strong dominance we mean that $d \approx 1$ such that terms of order $\mathcal{O}(\delta^2)$ can be neglect. If the modifier locus is fixed and codes for random mating, the same argument as in Section S1.3 yields the existence of a globally stable polymorphic equilibrium. It is approximately given by

$$\begin{aligned}
\hat{p}_1 &= \frac{1}{2} + \frac{\left((4-3\sqrt{2})c + (-2+\sqrt{2})s\right)}{4c}\delta + \mathcal{O}(\delta^2),\\
\hat{p}_2 &= \sqrt{2} - 1 + \frac{\left((-7+5\sqrt{2})c + (3-2\sqrt{2})s\right)}{2c}\delta + \mathcal{O}(\delta^2),\\
\hat{p}_3 &= \frac{3}{2} - \sqrt{2} + \frac{(3-2\sqrt{2}) + c\big(-3+2\sqrt{2}+(-7+5\sqrt{2})\big)}{2\,c}\delta + \mathcal{O}(\delta^2).
\end{aligned} \tag{S22}$$

Moreover we have

$$\phi = \frac{(-161564 + 114243\sqrt{2})(c-s)\delta}{19601 - 13860\sqrt{2} + (-66922 + 47321\sqrt{2})(c-s)\delta} + \mathcal{O}(\delta^2) \tag{S23}$$

$$= (3\sqrt{2} - 4)(c-s)\,\delta + \mathcal{O}(\delta^2) + \mathcal{O}(s^2). \tag{S24}$$

Since $3\sqrt{2} > 4$, and it follows that modifiers increasing assortment can invade if $c > s$.

S1.5 Intermediate dominance and complete assortment

It is easily verified that only homozygotes exist for complete assortment for any level of dominance, i.e., $\hat{p}_1 + \hat{p}_3 = 1$. Moreover, the equilibrium condition simplifies to $W_1 = W_3 = \overline{W}$. This condition implies that the only polymorphic equilibrium is the symmetric equilibrium $\hat{p}_1 = \hat{p}_3 = \frac{1}{2}$. To determine the local stability of this equilibrium we need to derive the Jacobian matrix. This can be done similar as in [4]. From this it is easily verified that the symmetric polymorphic equilibrium is locally asymptotically stable. Moreover, it is easily verified that the monomorphic equilibria are unstable (cf. [4]).

Consider an assortment modifier that leads to the mating probabilities

$$\pi(g,h) = \begin{cases} 1 & \text{if } Z_g = Z_h\,,\\ \xi_1 & \text{if } |Z_g - Z_h| = \delta\,,\\ \xi_2 & \text{if } |Z_g - Z_h| = 2-\delta\,,\\ \xi_3 & \text{if } |Z_g - Z_h| = 2\,. \end{cases} \tag{S25}$$

Because we assume $0 \le d < 1$, we can make the natural assumption $0 \le \xi_3 \le \xi_2 \le \xi_1 < 1$, which will hold as long as π is a monotone decreasing function of the differences between trait values.

Then at $\hat{p}_1 = \hat{p}_3 = \frac{1}{2}$ (S1) simplifies to

$$U = \begin{pmatrix} \alpha_1 & \eta\beta_1 r & \eta\beta_1(1-r) & 0\\ 0 & \eta\beta_1(1-r) & \eta\beta_1 r & 1-\alpha_1\\ 1-\alpha_1 & \eta(\frac{1}{2}-\beta_1)r & \eta(\frac{1}{2}-\beta_1)(1-r) & 0\\ 0 & \eta(\frac{1}{2}-\beta_1)(1-r) & \eta(\frac{1}{2}-\beta_1)r & \alpha_1 \end{pmatrix}, \tag{S26}$$

where $\alpha_1 = \frac{2+\xi_3}{2(1+\xi_3)}$, $\beta_1 = \frac{\xi_1}{2(\xi_1+\xi_2)}$, and $\eta = \frac{W_5}{W_1}$. Clearly, we have $\frac{1}{2} \leq \alpha_1 \leq 1$ and $\frac{1}{4} \leq \beta_1 \leq \frac{1}{2}$.

Unfortunately, in this case we cannot provide explicit expressions for the eigenvalues. However, we can prove that all eigenvalues have absolute value smaller than one.

Let $P(x)$ denote the characteristic polynomial of U. Clearly, $P(x)$ is a polynomial of degree four in x, with leading coefficient 1. We have

$$\begin{aligned} P(1) &= \frac{1}{4}(2-\eta)\big(2-\eta(1-2r)\big)(1-\alpha_1)^2 \\ P(-1) &= \eta(1-r)(1-\alpha_1)\alpha_1 + (1+\alpha_1)^2 + \frac{1}{4}\eta^2(1-2r)(1+\alpha_1-4\beta)(\alpha_1+4\beta-1)\,. \end{aligned}$$

Furthermore, note that

$$0 < \eta = \frac{W_5}{W_1} = 1 + d\frac{(s-c)(2-d)}{4-s+5c} \leq 1 + \frac{2s}{4-s} \leq 1 + \frac{2}{15} < 2\,.$$

Hence, for $\alpha_1 \neq 1$ we have $P(1) > 0$ and $P(-1) > 0$. Moreover, solving $P''(x) = 0$ yields the two solutions

$$q_{1,2} = \frac{A \pm B}{24}\,,$$

$$\begin{aligned} A &= 3\eta(1-r) + 12\alpha_1 \leq 15\,, \\ B &= \sqrt{3}\sqrt{16\alpha^2 + 8\eta(1-r)(2-3\alpha_1) + \eta^2(3(1-r)^2 - 16(1-2r)\beta_1 + 32(1-2r)\beta_1^2}\,. \end{aligned}$$

We see that $q_1 < 1$ by verifying $(24-A)^2 - B^2 > 0$, whereas $q_2 > -1$ follows from $(24+A)^2 - B^2 > 0$. Hence, $-1 < q_2 < q_1 < 1$. Thus, $P(x)$ is strict convex for $x \notin [-1,1]$. Since $P(1) > 0$ and $P(-1) > 0$, all real roots of P within $(-1,1)$. If $\alpha_1 \neq 1$, $\beta_1 \neq \frac{1}{2}$ and $r \neq 0$, U is irreducible such that according to the leading eigenvalue λ is real according to the Perron-Frobenius theorem. Hence, if U is irreducible $|\lambda| < 1$, i.e., the modifier cannot invade. Since the roots of P are continuous in the coefficients, we also have $|\lambda| < 1$ for $\beta \to \frac{1}{2}$, or $r \to 0$. This argument applies also for $\alpha_1 \to 1$. However, since we have $P(1) = 1$ for $\alpha_1 = 1$, we have $|\lambda| = 1$, i.e., the modifier is neutral.

S1.6 Complete dominance and complete assortment

As in Section S1.5, $\hat{p}_1 = \hat{p}_3 = 1/2$ is the only polymorphic equilibrium. Moreover, it can be shown that this equilibrium is stable but not asymptotically stable, and that both monomorphic equilibria are unstable.

Now, assume additionally complete dominance of one of the alleles. Without loss of generality we assume $d = 1$. Assume a modifier that leads to the mating probabilities

$$\pi(g,h) = \begin{cases} 1 & \text{if } Z_g = Z_h\,, \\ \xi & \text{if } |Z_g - Z_h| = 2\,, \end{cases} \tag{S27}$$

where we do not need to specify ξ in detail. At $\hat{p}_1 = \hat{p}_3 = \frac{1}{2}$ (S1) simplifies to

$$U = \begin{pmatrix} \alpha_1 & \alpha_1 r & \alpha_1(1-r) & 0 \\ 0 & \alpha_1(1-r) & \alpha_1 r & 1-\alpha_1 \\ 1-\alpha_1 & (1-\alpha_1)r & (1-\alpha_1)(1-r) & 0 \\ 0 & (1-\alpha_1)(1-r) & (1-\alpha_1)r & \alpha_1 \end{pmatrix}\,, \tag{S28}$$

where $\alpha_1 = \frac{2+\xi}{2(1+\xi)}$. Straightforward calculation yields the eigenvalues

$$\lambda_1 = 0, \;\; \lambda_2 = 1, \;\; \lambda_{3,4} = \frac{2\alpha_1 - r \pm \sqrt{r^2 - 8r(1-\alpha_1)^2 + 4(1-\alpha_1)^2}}{2} \, .$$

It is easily verified that $-1 \leq \lambda_{3,4} \leq 1$, so that the leading eigenvalues equals one. Hence, modifiers decreasing assortment are neutral.

It should be mentioned that we can also study a modifier decreasing the degree of dominance. Form eqs. (B2) to (B6) in [5], it becomes clear that a modifier decreasing dominance is also selectively neutral.

S1.7 Assortment vs. dominance

The invasion fitness of a modifier that induces an arbitrary degree of dominance in a randomly mating population is derived in [5]. The leading eigenvalue of the linearized transition matrix for a rare dominance modifier with effect d is given by

$$\lambda_d = 1 + d^2 \frac{(c-s)}{2+(2c-s)}. \tag{S29}$$

For an assortment modifier with effect $\tilde{a} \ll 1$ in an initially randomly mating population (S12) implies

$$\lambda_{\tilde{a}} = 1 + \tilde{a}\frac{c-s}{8} + \mathcal{O}\big(\tilde{a}^2\big). \tag{S30}$$

If the modifier effects d and $\tilde{a}$ go to 0, λ_d and $\lambda_{\tilde{a}}$ behave qualitatively differently. Because $\lambda_d = 1 + \mathcal{O}(d^2)$ and $\lambda_{\tilde{a}} = 1 + \mathcal{O}(\tilde{a})$, the strength of selection for a dominance modifier decreases faster than the strength of selection for an assortment modifier.

References

1. Matessi C, Gimelfarb A, Gavrilets S (2001) Long-term buildup of reproductive isolation promoted by disruptive selection: How far does it go? Selection 2 2: 41–64.

2. Schneider KA (2006) A multilocus-multiallele analysis of frequency-dependent selection induced by intraspecific competition. Journal of Mathematical Biology 52: 483-523.

3. Schneider KA (2008) Maximization principles for frequency-dependent selection I: the one-locus two-allele case. Theoretical Population Biology 74: 251–262.

4. Bürger R, Schneider KA, Willensdorfer M (2006) The conditions for speciation through intraspecific competition. Evolution 60: 2185–2206.

5. Peischl S, Schneider KA (2010) Evolution of dominance under frequency-dependent intraspecific competition in an assortatively mating population. Evolution 64: 561–582.

Tables

Table S1. Notation for genotypes in Section S1.

Genotype	$\frac{\mathscr{A}_1\mathscr{A}_1}{\mathscr{M}_1\mathscr{M}_1}$	$\frac{\mathscr{A}_1\mathscr{A}_2}{\mathscr{M}_1\mathscr{M}_1}$	$\frac{\mathscr{A}_2\mathscr{A}_2}{\mathscr{M}_1\mathscr{M}_1}$	$\frac{\mathscr{A}_1\mathscr{A}_1}{\mathscr{M}_1\mathscr{M}_2}$	$\frac{\mathscr{A}_1\mathscr{A}_2}{\mathscr{M}_1\mathscr{M}_2}$	$\frac{\mathscr{A}_1\mathscr{A}_2}{\mathscr{M}_2\mathscr{M}_1}$	$\frac{\mathscr{A}_2\mathscr{A}_2}{\mathscr{M}_1\mathscr{M}_2}$	$\frac{\mathscr{A}_1\mathscr{A}_1}{\mathscr{M}_2\mathscr{M}_2}$	$\frac{\mathscr{A}_1\mathscr{A}_2}{\mathscr{M}_2\mathscr{M}_2}$	$\frac{\mathscr{A}_2\mathscr{A}_2}{\mathscr{M}_2\mathscr{M}_2}$
Trait value	Z_1	Z_2	Z_3	Z_4	Z_5	Z_6	Z_7	Z_8	Z_9	Z_{10}
Fitness	W_1	W_2	W_3	W_4	W_5	W_6	W_7	W_8	W_9	W_{10}
Frequency	p_1	p_2	p_3	p_4	p_5	p_6	p_7	p_8	p_9	p_{10}
